# Down-regulation of guanylate binding protein 1 causes mitochondrial dysfunction and cellular senescence in macrophages

**DOI:** 10.1038/s41598-018-19828-7

**Published:** 2018-01-26

**Authors:** Xiaoxue Qiu, Hong Guo, Junshu Yang, Yinduo Ji, Chia-Shan Wu, Xiaoli Chen

**Affiliations:** 1 Department of Food Science and Nutrition, College of Food, Agricultural and Natural Resource Sciences, University of Minnesota, Twin Cities, Minnesota USA; 2 Department of Veterinary Biomedical Sciences, College of Veterinary Medicine, University of Minnesota, Twin Cities, Minnesota USA; 3 Department of Nutrition and Food Science, Texas A&M University, College Station, TX USA

## Abstract

Macrophage polarization is tightly associated with its metabolic reprograming and immune dysfunction. However, the intracellular molecules/pathways that connect these alterations in inflammatory macrophages remain largely unidentified. Herein, we explored the role of guanylate binding protein 1 (Gbp1), an intracellular anti-microbial protein, in regulating polarization, metabolic reprogramming, and cellular aging of macrophages. We showed that Gbp1 expression in inguinal white adipose tissue is significantly decreased in high-fat diet -fed and aged mice. Gbp1 expression is significantly induced by IFNγ and LPS in macrophages but not adipocytes. Downregulation of Gbp1 expression causes macrophage polarization towards a pro-inflammatory phenotype. Gbp1 knockdown (Kd) macrophages have impaired mitochondrial respiratory function, which is further supported by down-regulation of genes encoding electron transport chain components and genes involved in fatty acid oxidation and mitochondrial function. Moreover, we observed Gbp1 is localized in both cytosol and mitochondrial fraction, and Gbp1 Kd macrophages display decreased mitophagy activity. More interestingly, Gbp1 Kd macrophages undergo senescence as evidenced by increased activation of AMPK-p53 pathway and positive staining of β-galactosidase. These observations suggest that Gbp1 may play an important role in protecting against mitochondrial dysfunction and preserving immune function of macrophages during inflammatory stress and aging.

## Introduction

Adipose tissue inflammation is a hallmark characteristic of obesity and an important contributing factor for the development of insulin resistance and metabolic disorders in obesity^1,2^. Macrophages that infiltrate into adipose tissue and polarize to pro-inflammatory phenotype play a key role in obesity-associated adipose tissue inflammation and insulin resistance. During obesity, infiltrated T-cells, particularly interferon gamma (IFNγ)-secreting T helper type 1 cells^3^, promote macrophage polarization leading to the phenotypic switch of macrophages from a non-inflammatory and regulatory M2 towards a pro-inflammatory M1 phenotype^4^. Mechanistically, macrophages activated with the elevation of lipopolysaccharide (LPS) and IFNγ in obesity acquire an inflammatory M1 phenotype, characterized by increased production of pro-inflammatory cytokines and reactive oxygen species (ROS). These cytokines and ROS target adipocytes to further exacerbate adipose tissue inflammation and dysfunction^5^.

In response to signals derived from microbes and inflamed or damaged tissues, macrophages undertake functional adaptation to maintain immune homeostasis in host protection against infection. In addition to M1 polarization, macrophages coordinately undergo transcriptional rewiring and metabolic re-programming. For instance, M1 polarized macrophages activated with LPS or IFNγ display a metabolic shift from oxidative phosphorylation towards aerobic glycolysis, while M2 macrophages stimulated by IL-4 have a lower rate of glycolysis and increased oxidative phosphorylation^6,7^. Several studies have attempted to elucidate the possible molecular mechanisms of metabolic changes in macrophages^7–11^. For example, IL4-activated macrophages have been shown to exhibit increased uptake and oxidation of fatty acids and mitochondrial biogenesis, which requires the IL-4/STAT6-dependent induction of peroxisome-proliferator-activated receptor-γ co-activator-1β (PGC-1β)^11^. Since the mitochondrion plays a key role in oxidative phosphorylation and aerobic metabolism, mitochondrial dysfunction is considered a critical determinant for metabolic switch in inflammatory macrophages^12^ and it can also be an inducer of cellular senescence and aging^13,14^. However, there still remain some key questions to be addressed regarding which and how intracellular molecules/pathways regulate macrophage polarization and metabolic re-programming.

Guanylate binding protein 1 (Gbp1) is a 65 kD GTPase belonging to dynamin superfamily and performs enzymatic roles by oligomerizing and interacting with membranes to regulate a variety of cellular functions, such as budding and fusion of transport vesicles. Gbp1 has an amino-terminal globular domain of the GTP binding region and an elongated carboxy-terminal region containing a series of amphipathic α helices for protein-lipid and protein-protein interaction^15^. Kim *et al*. provided a line of evidence from *in vivo* studies showing that Gbp1 is critical for innate immunity; Gbp1-deficient mice were more susceptible to infection when challenged with *Mycobacterium bovis* or *Listeria monocytogenes*^16^. This phenotype has been attributed to the role of Gbp1 in transporting autophagic machinery to the pathogen containing vacuoles (PCVs). Additionally, Gbp1 has been shown to directly translocate to and eliminate PCVs via triggering cell-autonomous immune responses^17^. Gbp1 expression can be largely induced by IFNγ in macrophages, endothelial and epithelial cells^16,18,19^. A previous study has demonstrated a role of Gbp1 in maintaining intestinal homeostasis. Upon IFNγ stimulation, Gbp1 is expressed in intestinal epithelial cells to prevent cell apoptosis while promote cell proliferation^20^. Most of previous studies primarily focused on the role of Gbp1 in regulating innate immunity of macrophages to defend against pathogen infections. Little is known about the involvement of Gbp1 in regulating polarization, metabolic programing, and cellular aging of macrophages.

In this study, we tested the hypothesis that Gbp1 plays a role in regulating immunometabolism and senescence of macrophages. We found that Gbp1 was mainly expressed in macrophages, but not adipocytes in response to IFNγ/LPS stimulation; Gbp1 expression was significantly decreased in inguinal white adipose tissue (iWAT) of high-fat diet (HFD)-fed and aged mice. We also observed that downregulation of Gbp1 in macrophages resulted in M1 polarization and impairment of mitochondrial respiratory function possibly via disrupting mitophagy activity. Moreover, Gbp1 Kd macrophages displayed dampened glycolysis and exhibited senescence-associated secretory phenotype (SASP). These observations suggest that Gbp1 may play an important role in protecting against mitochondrial dysfunction and preserving immune function of macrophages during aging.

## Results

### Gbp1 expression in adipose tissue is regulated by HFD feeding and LPS stimulation

**Figure 1:**
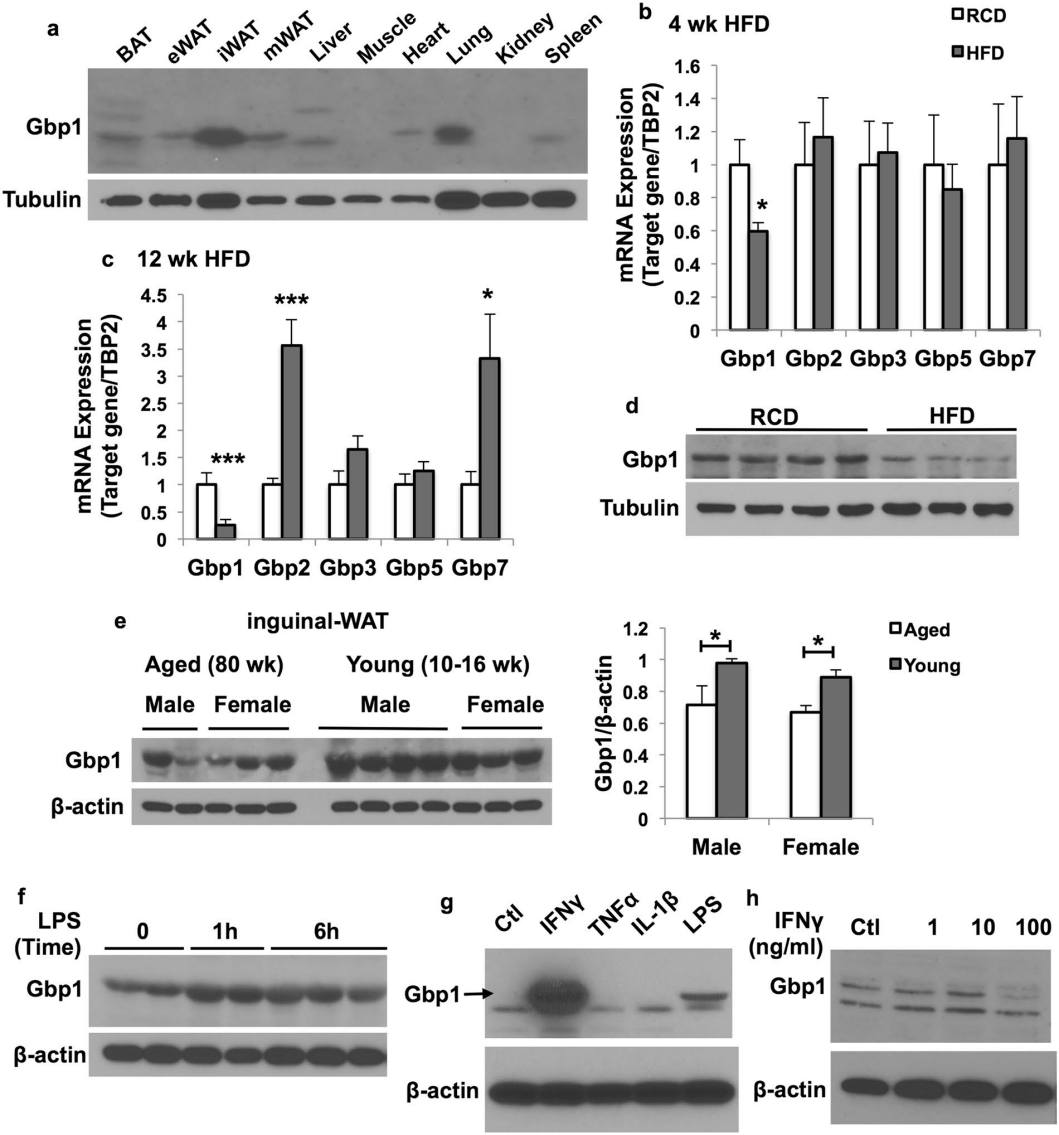
Regulation of Gbp1 expression in adipose tissue by HFD feeding and LPS stimulation. (**a**) Male mice were fed on a RCD for 12 weeks and sacrificed at the age of 16 weeks. Tissues were collected and homogenized in RIPA buffer. Gbp1 protein levels were determined by western blotting. (**b**,**c**) Male mice were fed on either a HFD or RCD for 4 (**b**) and 12 (**c**) weeks, respectively. Gene expression levels of Gbp isoforms in iWAT were detected by qPCR (n = 6). The values of gene expression are mean ± SEM. *P < 0.05, ***P < 0.001 versus RCD-fed mice. (**d**) Gbp1 protein levels in iWAT were compared between RCD- and 12-week HFD-fed mice. (**e**) Gbp1 protein expression levels in iWAT of RCD-fed male and female mice at the age of 80 weeks (aged) or 10–16 weeks (young) were detected by western blotting (left panel) and quantified by ImageJ (right panel). (**f**) Gbp1 protein expression in iWAT of RCD-fed male mice at the age of 16 weeks receiving intraperitoneal injection of LPS at the dose of 0.3 mg/kg. (**g**) Gbp1 protein expression in RAW 264 macrophages treated for 24 hours with various pro-inflammatory cytokines, *Ctl*: Control. (**h**) Gbp1 protein expression in 3T3-L1 adipocytes treated with various doses of IFNγ (1, 10, 100 ng/ml) for 24 hours. *Ctl*: Control. Full-length of all blots are provided in Supplementary Fig. 5.

First, we determined the tissue distribution of Gbp1 expression in regular chow diet (RCD)-fed male mice at 16 weeks of age. We found that Gbp1 was expressed at a relatively higher level in inguinal white adipose tissue (iWAT) and lung compared to other fat depots and tissues (Fig. 1a). Next, we assessed the regulation of *Gbps* (*Gbp1*, *Gbp2*, *Gbp3*, *Gbp5 and Gbp7*) expression in iWAT by HFD feeding. As shown in Fig. 1b,c, mRNA expression levels of *Gbp1* were significantly decreased in iWAT of mice after both 4 weeks and 12 weeks of HFD feeding compared to those in mice fed on a RCD. However, mRNA expression levels of other Gbp isoforms, including Gbp2, Gbp3, Gbp5 and Gbp7, were not significantly altered in iWAT of mice fed on a HFD for 4 weeks, while gene expression of Gbp2 and Gbp7 were significantly upregulated upon 12 weeks of HFD feeding (Fig. 1b,c). Consistently, protein levels of Gbp1 were also decreased in iWAT of mice fed on a HFD for 12 weeks compared to RCD-fed mice (Fig. 1d). Interestingly, we found Gbp1 protein levels were decreased in iWAT of aged male and female mice compared to young controls (Fig. 1e), suggesting that Gbp1 may play a role in regulating aging and cellular senescence. Moreover, Gbp1 protein levels were increased in iWAT in mice after one-hour LPS treatment, without a further increase upon 6-hour LPS stimulation (Fig. 1f). All the above data indicates that Gbp1 expression in iWAT is regulated by metabolic stress (HFD), aging and inflammatory stimulation.

To determine the cell types in adipose tissue that express Gbp1 in respond to HFD and inflammatory stimulation, we determined the regulation of Gbp1 protein expression by cytokines in adipocytes and macrophages, respectively. Gbp1 protein expression levels were very low in both 3T3-L1 adipocytes and Raw 264 macrophages under the basal condition (Fig. 1g,h). In macrophages, Gbp1 protein expression was strongly induced by IFNγ treatment and to a less extent by LPS, but not tumor necrosis factor alpha (TNFα) and interleukin-1beta (IL-1β) (Fig. 1g). IFNγ had no effect on Gbp1 expression in 3T3-L1 adipocytes (Fig. 1h). Together, these data indicate that IFNγ and LPS are the potent inducers of Gbp1 expression; macrophages are the primary Gbp1-expressing and IFNγ/LPS responsive cells in adipose tissue.

### Downregulation of Gbp1 causes M1 polarization and reduced immune function of macrophages

**Figure 2:**
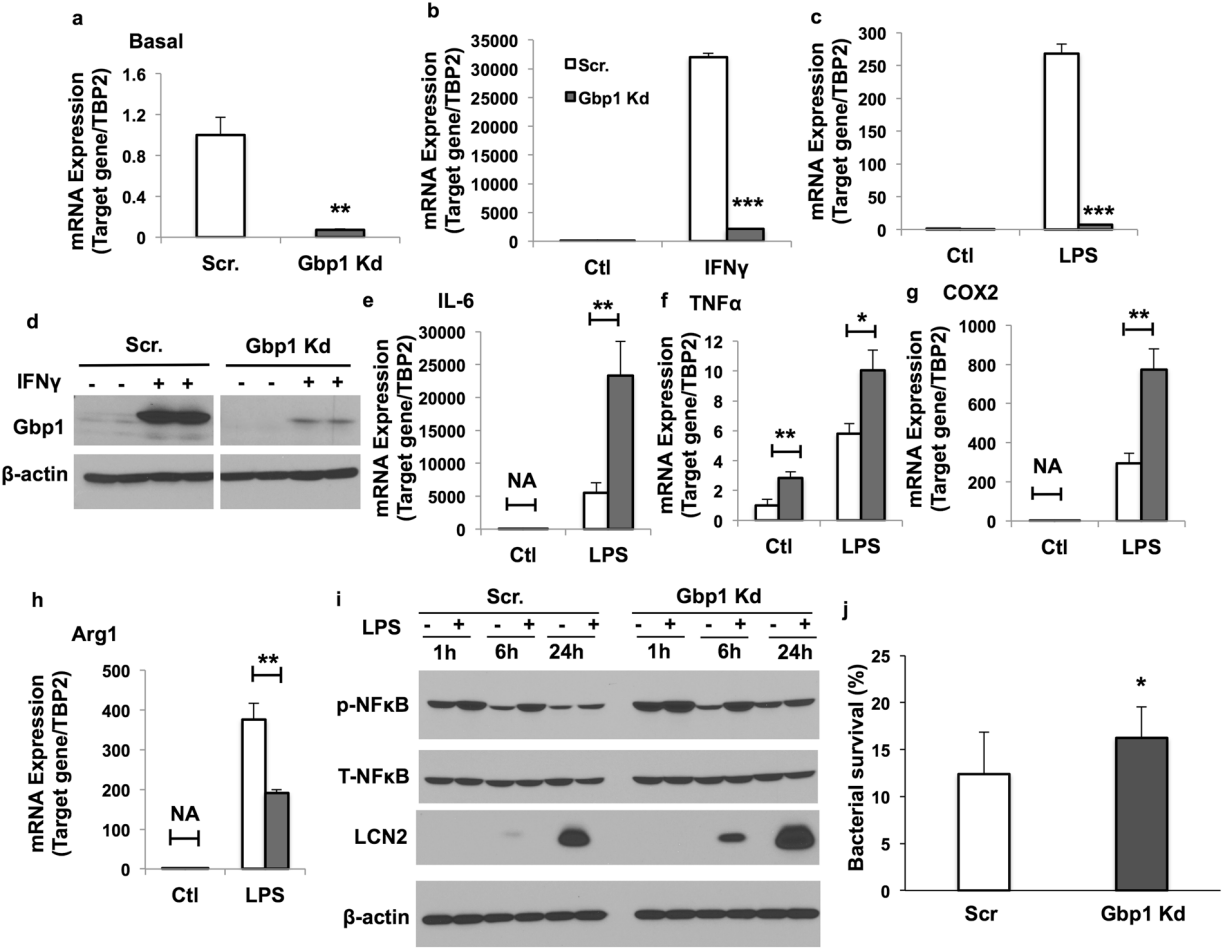
Effect of Gbp1 downregulation on M1 polarization of macrophages. (**a**–**d**) Gbp1 gene and protein expression levels in Scr and Gbp1 Kd macrophages treated with IFNγ (10 ng/ml) and LPS (1 μg/ml) for 24 hours. For panel d, samples were run in the same gels under the same experimental conditions while images of western blots displayed in cropped format. Full-length blots are provided in Supplementary Fig. 6. (**e**–**h**) mRNA expression levels of pro-inflammatory (IL-6, TNFα and COX2) and anti-inflammatory (Arg1) cytokines in Scr and Gbp1 Kd macrophages in response to 24 hour stimulation of LPS (1 μg/ml). The results are presented as mean ± SEM (n = 3). (**i**) NFκB phosphorylation and Lcn2 protein levels in macrophages treated with LPS (1 μg/ml) for 1, 6 and 24 hours. Full-length blots are provided in Supplementary Fig. 6. (**j**) Bacterial survival in macrophages treated with *S*. *aureus* for 1 hour. The values of bacterial survival represent the average from three independent experiments and each experiment was conducted with triplicate. Date are presented as mean ± SEM. *P < 0.05, **P < 0.01, ***P < 0.001; versus Scr cells.

To investigate the role of Gbp1 in the regulation of macrophage polarization and function, we established stable Gbp1 Kd macrophage cell lines using the pLKO.1-Puro lentivector system sub-cloned with Gbp1-siRNA sequence. Gbp1 gene expression was successfully silenced as indicated by markedly decreased mRNA and protein expression levels of Gbp1 under the IFNγ- or LPS-stimulated condition (Fig. 2a–d). After 24-hour treatment with LPS/IFNγ, Gbp1 Kd macrophages expressed significantly higher mRNA levels of pro-inflammatory cytokines, including IL-6, TNFα and COX2 (Fig. 2e–g and Supplementary Fig. S1), while lower levels of anti-inflammatory marker Arginase 1 (Arg1) (Fig. 2h and Supplementary Fig. S1) compared to Scr macrophages. TNFα mRNA expression levels were also significantly higher in Gbp1 Kd macrophages under the basal condition (Fig. 2f). Moreover, Gbp1 Kd macrophages had significantly increased NFκB phosphorylation under both basal and LPS/IFNγ treated conditions when compared to Scr cells (Fig. 2i and Supplementary Fig. S1). We also saw increased protein levels of lipocalin 2 (Lcn2), a good indicator of pro-inflammatory state in Gbp1 Kd macrophages (Fig. 2i). All the data together indicate that Gbp1 Kd macrophages undergo M1 polarization. Next, we performed bacterial killing assay to assess the immune function of Gbp1 Kd macrophages. To do that, we treated Scr and Gbp1 Kd macrophages with *Staphylococcus aureus* (*S*. *aureus*) for 1-hour infection. We found that the survival rate of *S*. *aureus* was significantly higher in Gbp1 Kd macrophages than Scr cells (Fig. 2j), suggesting that Gbp1 Kd macrophages have reduced ability to eliminate bacterial pathogens.

### Downregulation of Gbp1 impairs mitochondrial function in macrophages

**Figure 3:**
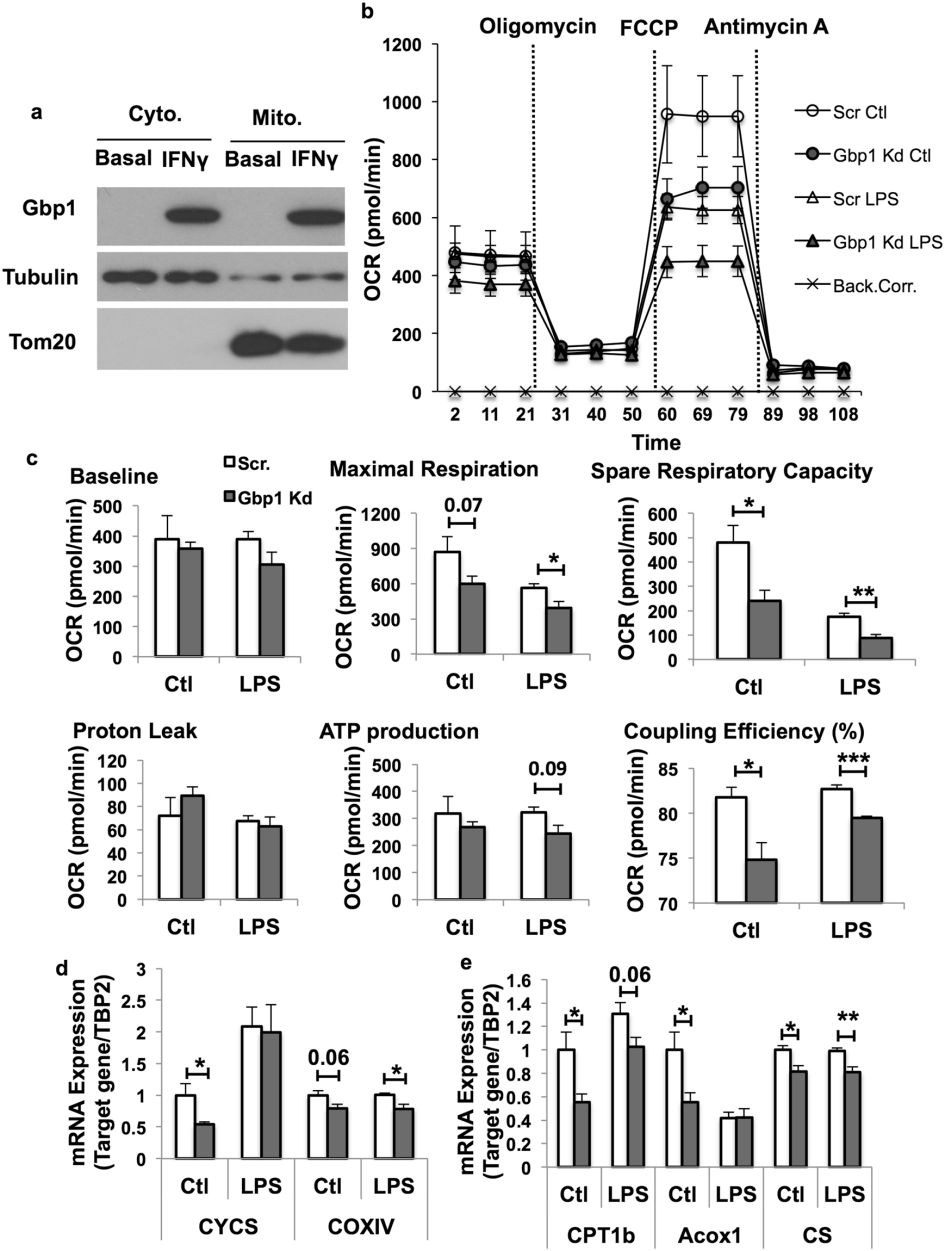
Effect of Gbp1 downregulation on mitochondrial function in macrophages. (**a**) Gbp1 protein levels in mitochondrial fraction of Raw 264 macrophages treated with 10 ng/ml IFNγ for 24 hours. Full-length blots are provided in Supplementary Fig. 7. (**b**,**c**) OCR in Scr and Gbp1 Kd macrophages treated with LPS (100 ng/ml) for 4 hours, n = 5. (**d**,**e**) mRNA expression levels of genes encoding electron transport chain components CYCS and COXIV, CTP1b, Acox1, and CS in Scr and Gbp1 Kd macrophages treated with 100 ng/ml LPS for 4 hours, n = 3. Data are presented as mean ± SEM. *P < 0.05, **P < 0.01, ***P < 0.001 versus Scr cells. Back.Corr.: Background Correction.

Metabolic reprogramming has been tightly correlated with macrophage polarization and function^21^. For instance, pro-inflammatory M1 macrophages display a metabolic transition towards aerobic glycolysis^7,22^. Since the mitochondrion plays a key role in this transition, we sought to determine if downregulation of Gbp1 impacts mitochondrial function. First, we isolated mitochondrial fraction from macrophages treated with and without IFNγ for 24 hours to determine the mitochondrial localization of Gbp1. We found that Gbp1 protein was distributed in both cytosol and mitochondrial fractions under the IFNγ-stimulated condition (Fig. 3a). Second, we performed a cellular respiratory assay using the Seahorse XF Analyzer to assess mitochondrial function in Gbp1 Kd macrophages. Our results showed that Gbp1 Kd macrophages had significantly decreased oxygen consumption rate (OCR) compared to Scr cells (Fig. 3b and Supplementary Fig. S2). The baseline respiratory rate was not different between Scr and Gbp1 Kd macrophages after 4-hour LPS stimulation (Fig. 3c), but significantly decreased in Gbp1 Kd cells after 16-hour IFNγ stimulation compared to Scr cells (Supplementary Fig. S2). Both LPS- and IFNγ-induced impairment in mitochondrial respiration was more pronounced in Gbp1 Kd macrophages, as demonstrated by a significant reduction in maximal respiration rate, coupling efficiency, and spare respiratory capacity (SRC) (Fig. 3c and Supplementary Fig. S2). While no significant difference in proton leak and ATP production was observed between Scr and Gbp1 Kd macrophages under the LPS stimulation, ATP production was significantly lower in Gbp1 Kd macrophages compared to Scr cells under the IFNγ treatment (Fig. 3c and Supplementary Fig. S2). These results suggest that Gbp1 Kd macrophages may have impaired mitochondrial function. Finally, our results showed that the mRNA expression levels of mitochondrial genes cytochrome c oxidase (COXIV) and cytochrome C (CYCS) were significantly decreased in Gbp1 Kd macrophages under the basal condition (Fig. 3d and Supplementary Fig. S2). The mRNA expression level of *COXIV* was also significantly downregulated in Gbp1 Kd macrophages treated with either LPS or IFNγ (Fig. 3d and Supplementary Fig. S2). Additionally, the mRNA expression levels of genes that regulate fatty acid metabolism such as carnitine palmitoyltransferase 1b (*CPT1b*), *Acox1*, and citrate synthase *(CS)* were significantly lower in Gbp1 Kd macrophages compared to Scr cells (Fig. 3e), suggesting a decrease in fatty acid oxidation and impaired mitochondrial function.

### Downregulation of Gbp1 impairs mitophagy in macrophages

**Figure 4:**
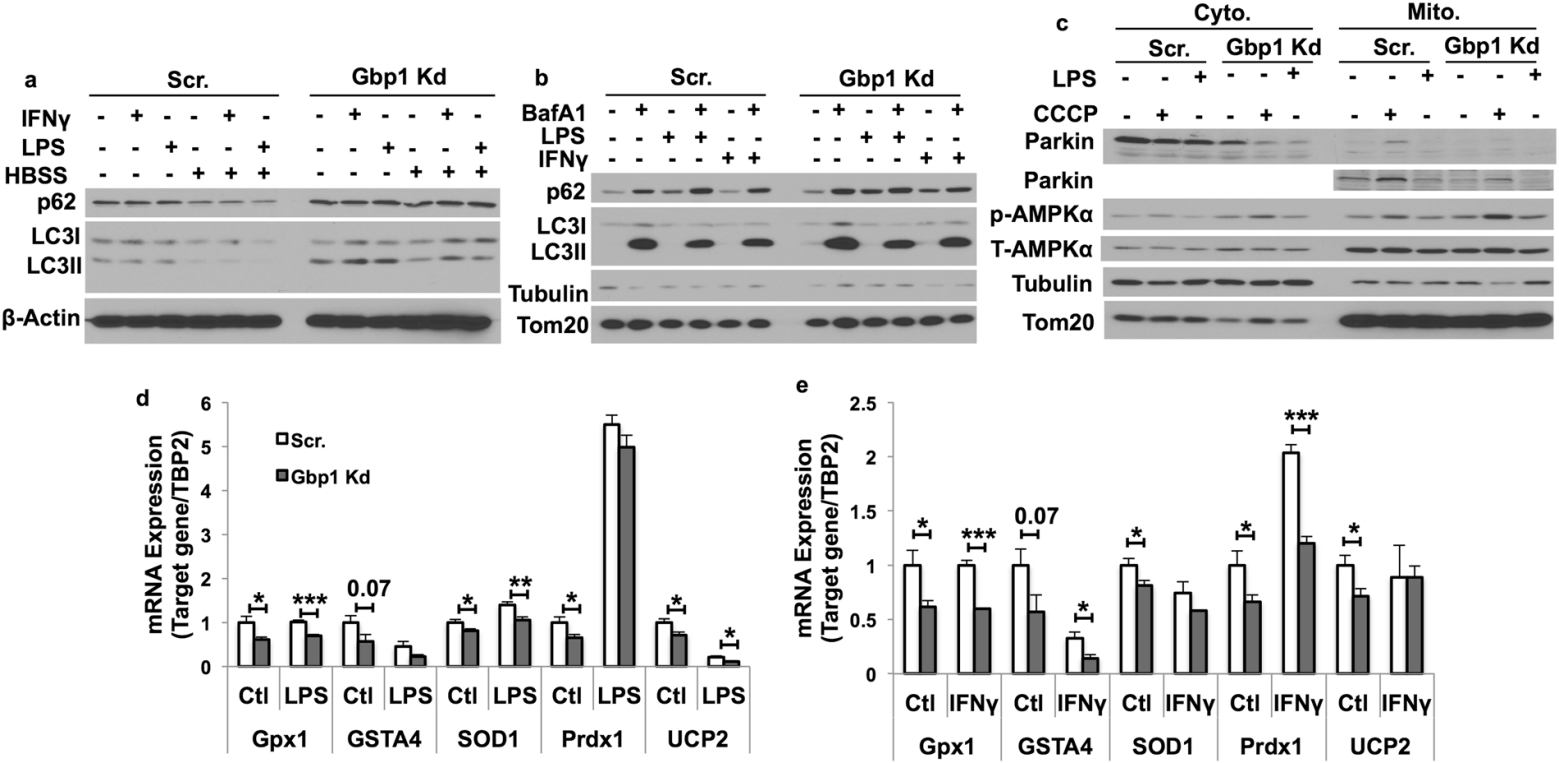
Effect of Gbp1 downregulation on mitophagy in macrophages. (**a**) Autophagic proteins p62 and LC3I/II levels in macrophages treated with LPS (1 μg/ml) or IFNγ (10 ng/ml) for 1 hour under fed or starvation conditions. (**b**) Autophagic protein levels in mitochondrial fraction of macrophages treated with LPS (100 ng/ml) or IFNγ (10 ng/ml) in the presence or absence BafA1 (100 nM) for 4 hours. (**c**) The levels of mitophagy related proteins in cytosolic and mitochondrial fractions isolated from macrophages treated with 100 ng/ml LPS or 10 μM CCCP for 4 hours. (**d**,**e**) mRNA expression levels of genes encoding anti-oxidative enzymes in macrophages treated with either 100 ng/ml LPS (**d**) or 10 ng/ml IFNγ (**e**) for 24 hours. The results are mean ± SEM. *P < 0.05, **P < 0.01, ***P < 0.001 versus Scr cells. HBSS: Hanks’ Balanced Salt Solution; CCCP: Carbonyl cyanide m-chlorophenylhydrazone. Full-length of all blots are provided in Supplementary Fig. 8.

Gbp1 is known to function as a regulator of autophagic machinery recruitment potentially through binding p62^16^. This led us to further examine the impact of Gbp1 deficiency on autophagy/mitophagy that may contribute to the mechanism for its role in regulating mitochondrial function. As we expected, there was a significant accumulation of the autophagic substrate p62 in Gbp1 Kd macrophages in the basal condition and the condition with LPS or IFNγ treatment for one hour (Fig. 4a). Moreover, one hour of nutrient deprivation (HBSS treatment) significantly decreased p62 and LC3 levels in Scr cells (Fig. 4a). However, this effect was blunted in Gbp1 Kd macrophages (Fig. 4a), suggesting impaired starvation-induced autophagy (Fig. 4a and Supplementary Fig. S3). Since Gbp1 is localized in mitochondria (Fig. 3a), we tested the hypothesis that Gbp1 may play a role in regulating mitophagy, a specific autophagy targeting damaged or dysfunctional mitochondria. We performed autophagic flux assay and examined autophagic protein levels in mitochondrial fractions isolated from Scr and Gbp1 Kd macrophages. BafA1 treatment led to a significant increase in both p62 and LC3II protein levels in mitochondrial fractions in Scr cells (Fig. 4b). Compared to Scr cells, LPS-treated Gbp1 Kd macrophages had higher levels of mitochondrial p62, but reduced response to BafA1 blockage in mitochondrial p62 accumulation, while mitochondrial LC3 levels were not significantly different between Scr and Gbp1 Kd cells under all different conditions (Fig. 4b). To provide direct evidence for the impact of Gbp1 on mitophagy activity, we treated macrophages with carbonyl cyanide m-chlo-rophenylhydrazone (CCCP), a mitochondrial membrane depolarizing drug, to induce mitochondrial stress and trigger mitochondrial damage-induced mitophagy^23^. When treated with CCCP, both cytosol and mitochondrial fractions of Gbp1 Kd macrophages displayed significantly lower levels of parkin protein, a protein that is selectively recruited to impaired mitochondria and promotes mitophagy^24^, compared to that of Scr macrophages. This suggests an impairment of mitophagy in Gbp1 Kd macrophages (Fig. 4c).

Furthermore, we assessed the status of oxidative stress as indicative of mitochondrial dysfunction. We found mRNA expression levels of genes encoding anti-oxidative enzymes, including glutathione peroxidase (GPX1), glutathione S-transferase alpha 4 (GSTA4), superoxide dismutase 1 (SOD1), peroxiredoxin 1 (PRDX1) and UCP2, were significantly downregulated in Gbp1 Kd macrophages compared to Scr cells under the basal and LPS-treated condition (Fig. 4d). Similarly, decreased mRNA levels of *GPX1*, *GSTA4*, *and PRDX1* were also observed in Gbp1 Kd macrophages with IFNγ stimulation (Fig. 4e). This data further supports the impaired mitophagy in Gbp1 kd macrophages.

### Downregulation of Gbp1 diminishes LPS-induced glycolysis in macrophages

**Figure 5:**
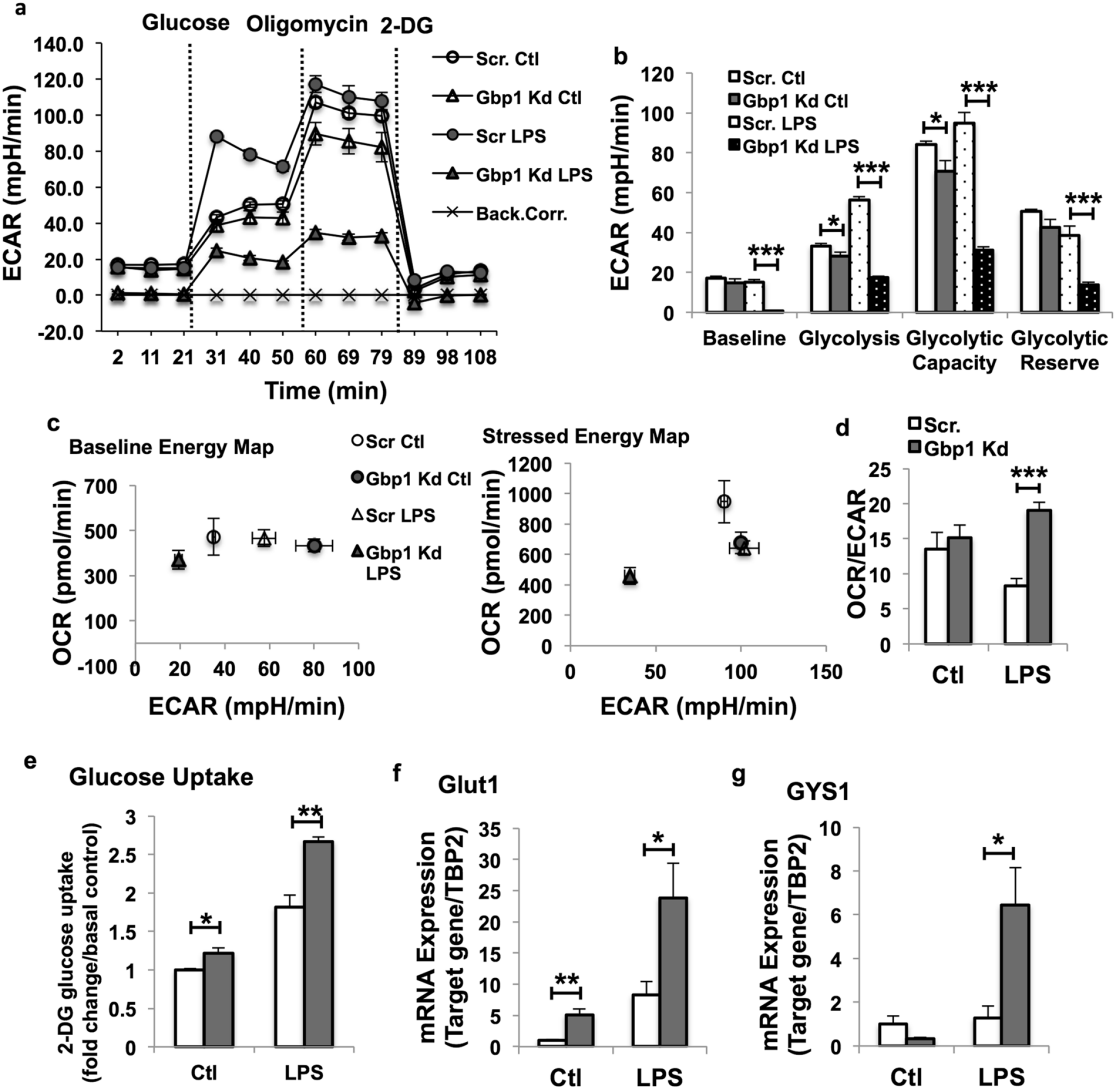
Effect of Gbp1 downregulation on LPS-induced glycolysis in macrophages. (**a**–**d**) ECAR in Scr and Gbp1 Kd macrophages treated with LPS (100 ng/ml) for 4 hours (n = 5). Back.Corr.: Background Correction. (**e**) 2-Deoxy glucose uptake in Scr and Gbp1 Kd macrophages under basal and LPS-treated conditions. Three independent experiments were performed. Error bars represent standard error. (**f**,**g**) mRNA expression levels of genes involved in glucose uptake and glycogen synthesis in Scr and Gbp1 Kd macrophages. The data for gene expression are presented as mean ± SEM; the experiment was repeated 2–3 times. *P < 0.05, **P < 0.01, ***P < 0.001; versus Scr cells.

Our results above have demonstrated that reducing Gbp1 expression polarizes M1 macrophages and induces mitochondrial dysfunction. Next, we determined whether and how Gbp1 promotes reprograming of glucose metabolism. To that end, macrophages were exposed to glycolytic stress compounds in the following order: glucose, oligolmycin and 2-deoxyglycose during the real-time ECAR measurement using the Seahorse XF Analyzers. Unexpectedly, the baseline ECAR levels were significantly lower in LPS-treated Gbp1 Kd macrophages compared to LPS-treated Scr cells, while the baseline ECAR was not altered between these two genotypes under the control condition (Fig. 5a,b). In the presence of glucose, Gbp1 Kd macrophages had lower levels of ECAR than Scr cells under the basal condition (Fig. 5b). Upon LPS treatment, Scr macrophages underwent a dramatic increase in glycolytic rate. In contrast, Gbp1 Kd macrophages exhibited diminished response to LPS-induced glycolysis and had decreased ECAR compared to untreated control cells (Fig. 5a,b). Oligomycin was added to inhibit mitochondrial ATP synthase activity, which leads to a shift of the energy production to glycolysis and induces the maximal glycolytic capacity. Similarly, Gbp1 Kd macrophages displayed a significant decrease in glycolytic capacity compared to Scr cells under both control and LPS-treated conditions (Fig. 5a,b). Lastly, glycolytic reserve, which indicates the ability of a cell to perform glycolysis in response to an energetic demand, was also significantly reduced in Gbp1 Kd macrophages only under the LPS-stimulated condition (Fig. 5a,b). Additionally, the results of energy map showed that in response to LPS stimulation, Gbp1 Kd macrophages exhibited both lower OCR and ECAR compared to Scr cells under both basal and stressed conditions, and the ratio of OCR to ECAR was significantly higher in Gbp1 Kd macrophages under the LPS-treated condition (Fig. 5c,d). All the data indicates that Gbp1 Kd macrophages have lower glycolytic activity in the pro-inflammatory stress condition.

We then performed glucose uptake assay to address the question of whether the reduced glycolysis in Gbp1 Kd macrophages is due to less glucose taken up by these cells. We found that Gbp1 Kd macrophages indeed took up more glucose than Scr cells under both basal and LPS-treated conditions (Fig. 5e). Consistently, mRNA expression levels of *Glut1*, a glucose transporter expressed at a higher level in macrophages^9^, were significantly upregulated in Gbp1 Kd macrophages compared to Scr cells (Fig. 5f). Since glycolytic activity was reduced in LPS-treated Gbp1 Kd macrophages, we then investigated the metabolic destination of these uptaken glucose in Gbp1 Kd macrophages. A previous study reported that a pro-inflammatory phenotype is associated with enforced glycogen deposition in macrophages^25^. Interestingly, we observed mRNA expression levels of glycogen synthase 1 (*GYS1*) were significantly upregulated in Gbp1 Kd macrophages under the LPS-treated condition, but not the basal condition (Fig. 5g), suggesting that uptaken glucose may be pushed to the glycogen synthesis pathway instead of glycolysis in LPS-treated Gbp1 Kd macrophages.

### Downregulation of Gbp1 leads to cellular senescence of macrophages

**Figure 6:**
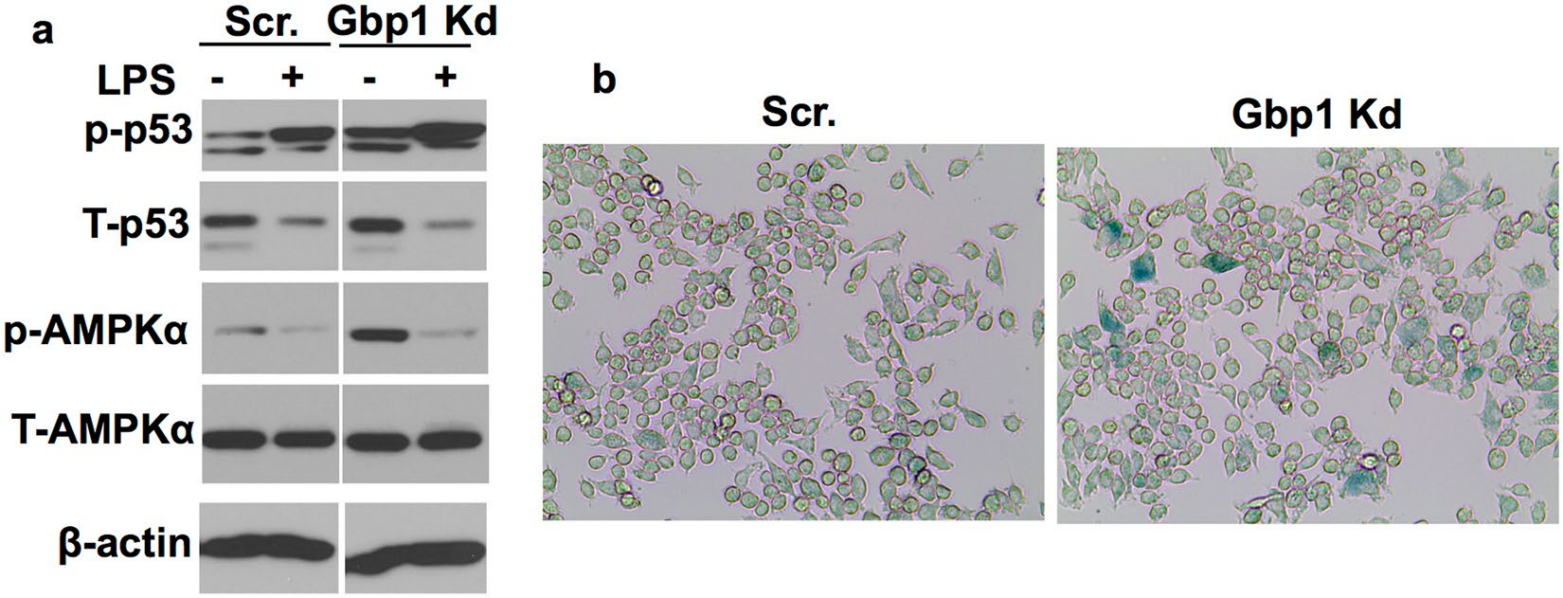
Effect of Gbp1 downregulation on cellular senescence in macrophages. (**a**) Phosphorylated p53 and AMPK levels in Scr and Gbp1 Kd macrophages treated with 100 ng/ml LPS for 24 hours. Samples were run in the gels under the same experimental conditions while images of western blots displayed in cropped format. Full-length blots are provided in Supplementary Fig. 9. (**b**) Representative β-gal staining of Scr and Gbp1 Kd macrophages under the basal condition. The values of protein expression are mean ± SEM. *P < 0.05 versus young mice. The blots of p-p53, T-p53, p-AMPKα, T-AMPKα and β-actin for Scr. and Gbp1 Kd macrophages in panel B were cropped from different parts of a same gel. White space was used between all the blots for Scr. and Gbp1 Kd cells.

Diminished response to LPS-induced glycolysis in Gbp1 Kd macrophages apparently contradicts the reprograming of glucose metabolism (increased glycolysis) that is often seen in M1 polarized macrophages^5^, but indicates that Gbp1 Kd macrophages may turn into a quiescent or senescent state. Given that downregulation of Gbp1 impairs mitochondrial function and autophagic process, both of which are closely associated with cellular senescence and aging^14,26,27^, we hypothesize that Gbp1 Kd macrophages may undergo aging and cellular senescence. Since Gbp1 is highly expressed in macrophages in response to LPS, a stimulus of senescence, we then examined the activity of a key signaling pathway (AMPK-p53) that is activated by mitochondrial dysfunction-associated senescence (MiDAS), as well as senescence markers in Gbp1 Kd macrophages. As shown in Fig. 6a, Gbp1 Kd macrophages displayed significantly higher levels of phosphorylated p53 and AMPK than Scr cells in the basal condition, and LPS treatment for 24 hours induced more p53 phosphorylation in Gbp1 Kd cells compared to Scr cells. Furthermore, we found there were an increased number of β-gal-stained macrophages in Gbp1 Kd cell cultures compared to Scr controls in the basal condition (Fig. 6b). These results strongly suggest that Gbp1 has a protective role against senescence of macrophages.

## Discussion

Macrophages play a key role in the development of chronic low-grade inflammation that occurs in multiple tissues, such as adipose tissue and liver during obesity. Understanding the factors that regulate macrophage polarization and function is crucial for developing a therapeutic approach to control tissue inflammation in obesity and its related metabolic complications. Gbp1, a 65 kD GTPase is known to have an important function in innate immunity; it has been widely shown to play a role against the replication of bacteria and parasites^16,28–30^. Gbp1 expression can be induced by bacterial infection; Gbp1 knockout mice display increased bacteria burdens in the spleen and liver^16^. LPS stimulation, which is increased during bacterial infection and metabolic stress, can also induce Gbp1 protein expression. This increase is likely a protective mechanism to eliminate the invasive bacteria and reduce the inflammatory stress. In this study, we found that Gbp1 expression is highly induced by IFNγ and LPS in macrophages but not adipocytes (Fig. 1g,h); Gbp1 protein expression is significantly downregulated in iWAT of HFD-fed and aged mice (Fig. 1b,d and e). This data suggests HFD feeding and aging fail to boost Gbp1 expression in iWAT, leading to the loss of the protective regulatory mechanism of Gbp1 in iWAT, which may be one of the possible mechanisms contributing to HFD- and age-induced adipose tissue inflammation.

Given that macrophages are the main Gbp1-expressing cell type in adipose tissue, and the proinflammatory cytokines IFNγ and LPS are the potent inducers of Gbp1 expression in macrophages (Fig. 1g), we focused on the role of Gbp1 in regulating macrophage polarization and function. It is well documented that macrophages play an important role not only in inflammatory responses against pathogen infections, but also chronic metabolic stress in obesity. Infiltration and M1 pro-inflammatory polarization of macrophages is a major contributor to adipose tissue inflammation during obesity. Numerous studies have been reported that metabolic pathways are altered during polarization in macrophages. M1 macrophages prefer aerobic glycolysis to oxidative phosphorylation. This metabolic reprogramming is similar to the Warburg effect that occurs in cancer cells displaying increased uptake and utilization of glucose mainly through glycolysis. On the other hand, changes in metabolism can also alter the differentiation and function of immune cells. For example, gain of Glut1 function or enforced glycogen storage led to the formation of pro-inflammatory polarized macrophages^9,25^. However, the molecular mechanisms of IFNγ/LPS-induced pro-inflammatory polarization accompanied by metabolic rewiring in macrophages remain elusive. To understand the role of Gbp1 in regulating polarization and metabolic profiles of macrophages, we established the stable Gbp1 Kd macrophage cell lines. Our results showed that in response to LPS/IFNγ stimulation, downregulation of Gbp1 causes upregulation of pro-inflammatory cytokines, enhanced NFκB activation, as well as increased Lcn2 levels (Fig. 2 and Supplementary Fig. S1), indicating that Gbp1 acts as an anti-inflammatory regulator during macrophage polarization. Interestingly, we found that Gbp1 is localized in both mitochondrial and cytosolic fraction (Fig. 3a). As a GTPase, Gbp1 function requires the catalytic activity of the GTPase domain, as well as homo- and hetero-oligomerization^31^. The binding affinity for endomembranes is conferred to Gbp1 upon isoprenylation on its C-termianl CAAX box^32^. Based on its structure and cellular location, we suspected Gbp1 may regulate mitochondrial function. As expected, Gbp1 Kd macrophages had impaired mitochondrial respiratory function under both the basal and inflammatory conditions (Fig. 3 and Supplementary Fig. S2).

Mitophagy, a specific type of macroautophagy is an important intracellular process for the clearance of damaged or dysfunctional mitochondria through Parkin-dependent ubiquitin conjugation and p62-mediated recognition. The normal function of this process is critical for mitochondrial quality control. Gbp1 exerts its anti-microbial role through binding with p62 and ubiquitin, promoting autophagic process to kill invasive pathogens in macrophages^16,29^. Our results demonstrated that downregulation of Gbp1 leads to a decrease in Parkin protein levels in the mitochondrial fraction, as well as accumulation of p62 and LC3 proteins, indicating that Gbp1 is critical for mitophagic process. Decreased mitophagic activity may cause accumulation of damaged mitochondria and mitochondrial dysfunction, leading to oxidative stress and inflammation in Gbp1 Kd macrophages. In fact, we saw the upregulation of proinflammatory genes such as IL-6 and TNFα (Fig. 2b and Supplementary Fig. S1), the downregulation of genes encoding anti-oxidative enzymes (Fig. 4d,e). This data suggests that downregulation of Gbp1 renders macrophages more sensitive to LPS-induced mitochondrial dysfunction, oxidative stress, and inflammation.

It is well known that pro-inflammatory phenotype M1 macrophages undergo metabolic re-programming typically characterized by increased glycolysis and decreased oxidative phosphorylation^7,9,21,25^. Interestingly, both aerobic glycolytic activity (Fig. 5a,d) and oxidative phosphorylation were significantly decreased in Gbp1 Kd macrophages despite increased glucose uptake (Fig. 5e,f). Since *GYS1* mRNA expression levels are increased under LPS stimulation, it is possible that the uptaken glucose may be favorably stored as glycogen in Gbp1 Kd macrophages (Fig. 5g). This speculation is supported by previous reports that increased glucose uptake and enhanced glycogen accumulation were seen in M1 polarized macrophages^9,25^. As shown in the results of energy map, Gbp1 Kd macrophages have both lower OCR and ECAR (Fig. 5c), suggesting that these cells may favor to enter the G_0_ cell cycle and be transformed into quiescent status. Quiescent macrophages are usually deactivated and in a steady state phenotype^33^. However, Gbp1 Kd macrophages have a pro-inflammatory phenotype (Fig. 2b,d and Supplementary Fig. S1), which doesn’t seem to support the quiescent state of Gbp1 Kd macrophages. Considering that increased production of pro-inflammatory cytokines (IL-6 and TNFα) and impaired mitochondrial function are commonly associated with SASP, we hypothesized that Gbp1 may function in regulating senescence. The AMPK-p53 is known as the main pathway that controls cell-cycle arrest and induces senescence^14,34^. We found Gbp1 Kd macrophages have increased levels of both p53 and AMPK phosphorylation (Fig. 6a), as well as senescence marker β-gal compared to Scr cells (Fig. 6b). Moreover, Gbp1 protein levels were decreased in iWAT of aged mice compared to young controls. These results together strongly suggest that downregulation of Gbp1 causes macrophage senescence. In the senescent state, Gbp1 Kd macrophages display SASP, but decreased glycolytic activity, which could be explained by increased p53 activation. Indeed, p53 plays an important role in the regulation of glycolysis by acting as an antagonist against the early stage of glycolysis^35,36^. From this perspective, increased p53 activation is likely to limit the glycolysis of Gbp1 Kd macrophages, leading to this specific scenario. However, an alternative mechanism for explaining this scenario cannot be ruled out.

**Figure 7:**
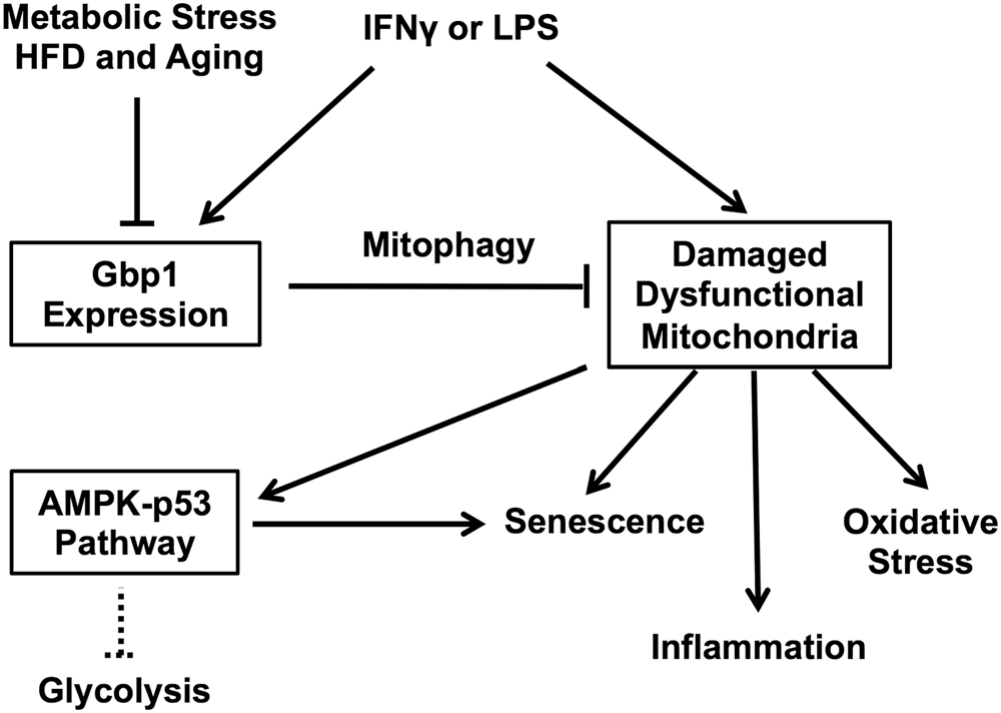
Schematic model for the role of Gbp1 in macrophages. Gbp1 expression is upregulated in response to the stimulation of pro-inflammatory cytokines (IFNγ and LPS) as a defensive mechanism during infection and inflammation. Gbp1 functions in eliminating dysfunctional mitochondria induced during inflammation via promoting mitophagic pathway. During chronic metabolic stress, such as HFD feeding and aging, Gbp1 is downregulated leading to mitochondrial dysfunction and subsequent activation of AMPK-p53 pathway and cellular senescence. Activation of p53 can also limit glycolytic activity of senescent cells.

In summary, our study suggests that Gbp1 is involved in mitophagy to clear damaged or dysfunctional mitochondria caused by pro-inflammatory cytokine stimulation (Fig. 7). Downregulaiton of Gbp1 reduces mitophagic activity, leading to mitochondrial dysfunction, oxidative stress, inflammation, and senescence during inflammatory stress (Fig. 7). We conclude that Gbp1 plays a protective role against inflammation-induced dysregulation of inflammatory response and metabolism in macrophages through promoting mitophagy to maintain mitochondrial function and prevent MiDAS. Decreased Gbp1 expression in iWAT of aged mice implies that Gbp1 may play a key role in preserving macrophage aging.

## Methods

### Animals

C57BL/6 mice were housed with a 12-hour light-dark cycle in a specific pathogen-free facility at the University of Minnesota. Male mice were weaned at the age of 4 weeks, followed by the feeding with a regular chow diet (RCD) or a HFD (fat calories: 60% lard; Bio-Serv F3282; New Brunswich, NJ) for 4 and 16 weeks, respectively. Animal handling followed the National Institutes of Health guidelines, and experimental procedures were approved by the University of Minnesota Animal Care and Use Committee.

### Lipopolysaccharide Injection in Mice

RCD-fed male C57BL/6 mice at the age of 16 weeks received intraperitoneal injection of lipopolysaccharide (LPS) at the dose of 0.3 mg/kg. Mice were then sacrificed after 1 or 6 hour(s) of LPS treatment. Inguinal WAT was collected for the measurement of Gbp1 protein levels.

### Cells

Raw 264 macrophages and 3T3-L1 fibroblasts were kindly provided by Dr. David A. Bernlohr at the University of Minnesota. Raw 264 macrophages were cultured in RPMI 1640 medium (Invitrogen, Carlsbad, CA) with 10% fetal bovine serum (FBS). To establish Gbp1 Kd and Scrambled (Scr) macrophages cell lines, the shRNA targeting *Gbp1* gene or nonspecific Scr were subcloned into lentivector pLKO.1. The selected oligomer targeting *Gbp1* was 5′-AGCAGATTGAAATGGAACGTA-3′. Transduction was then performed using lentivirus-carring shRNA in the macrophages as previously described^37^. Stable cell lines expressing the shRNA targeting Gbp1 and nonspecific target were selected by 2 μg/ml of puromycin (Sigma-Aldrich, Saint Louis, MO) for 2 days. 3T3-L1 fibroblasts were maintained in high-glucose DMEM medium (Invitrogen, Carlsbad, CA) and induced to differentiate into adipocytes as described previously^38^. Fully differentiated 3T3-L1 adipocytes were treated with IFNγ for 24 hours.

### RNA Isolation and Relative Quantitative RT-PCR

Total RNA was extracted from cell and tissue samples using TRIzol (Invitrogen, Carlsbad, CA). cDNA was synthesized using SuperScript II Reverse Transcriptase Kit (Invitrogen, Carlsbad, CA). Quantitative PCR was conducted using SYBR Green qPCR Master Mix (SABioscience, Frederick, MD) by StepOne Real-Time PCR system (Applied Biosystem Foster City, CA). ΔΔCt method was used to calculate the results. TATA box binding protein (TBP) gene was used as the internal reference gene. The primer sequences for amplifying target genes are provided in Supplementary Table S1.

### Western Blotting

Cell and tissue samples were lysed using RIPA buffer (Sigma-Aldrich, Saint Louis, MO) supplemented with protease inhibitor mixture (Roche Diagnostics). Protein concentrations were measured using the bicinchoninic acid (BCA) method. Equal amounts of proteins were loaded and separated on 10% SDS-PAGE, transferred to a nitrocellulose membrane and probed with indicated antibodies. Blots were visualized by enhanced chemiluminescence (Thermo Scientific, Rockford, IL). The following antibodies were used in this study: anti-Gbp1, anti- NFκB and anti-Tom20 (Santa Cruz); anti-phospho-NFκB, anti-phospho-p53, anti-p53, anti-phospho-AMPK, anti-AMPK, anti-Parkin, anti-p62, anti-LC3I/II, anti- β-actin and anti-tubulin (Cell Signaling Technology); anti-Lcn2 (R&D System, Minneapolis, MN).

### Mitochondrial Isolation

Macrophages were scraped in ice-cold isolation buffer (pH 7.4, 20 mM Tris-HCl, 1 mM EDTA, 0.2 mM EGTA) supplemented with protease inhibitor and lysed with 20 strokes of a glass-teflon homogenizer at 1600 rpm. Homogenates were centrifuged at 700 *g* for 10 to collect the supernatant, which was then centrifuged at 9000 *g* for 10 min to collect the supernatant (cytosolic) fraction. The pellet was re-suspended in RIPA lysis buffer supplemented with protease inhibitor mixture (Roche Diagnostics) as mitochondrial fraction.

### Cellular Respiratory Assay and Glycolysis Rate Measurement

An XF24 extracellular flux analyzer (Seahorse Biosciences) was applied to measure both cellular respiration and glycolysis rate according to the manufacture user guide. Macrophages were seeded on V7 microplates at a density of 500,000 cells per plate and incubated overnight. Cells were treated with either LPS (100 ng/ml) for 4 hours or IFNγ (10 ng/ml) for 16 hours. During the cellular respiratory assay, the cells were stimulated by the compounds in the following order: 2 μM oligomycin, 0.4 μM FCCP, and 4 μM antimycin A. Respiration rates were calculated as previously described^39^. According to Seahorse XF glycolysis stress kit user guide, culture medium was replaced with glucose-free XF assay buffer containing 2 mM glutamine after 4-hour LPS (100 ng/ml) stimulation. After one-hour incubation at 37 °C under the non-CO_2_ condition, cells were exposed to glycolysis stress compounds in the following order: 2 mg/ml glucose, 1 μM oligomycin, and 50 mM 2-deoxyglucose. Glycolysis rates were calculated as previously described^40^.

### Glucose Uptake

Glucose uptake was measured using [^3^H]2-DG as described previously^8^. Briefly, RAW 264 macrophages were seeded at the density of 500,000 cells/well, and allowed to adhere for 2 h at 37 °C. Cells were treated with or without LPS (100 ng/ml) for 4 h in RPMI 1640 medium with 10% FBS. Cells were then washed twice with warm PBS, and preincubated for 10 min in glucose-free DMED without FBS. Glucose (100 μM) and [^3^H]2-DG (0.5 μCi/ml) were added, and then cells were incubated in 37 °C without CO_2_ for 5 min. Glucose uptake was stopped by the addition of 100 μl of ice-cold PBS buffer with 5.2 mg/ml KCN for 3 min. Cells were then washed twice with cold PBS containing 20 mM D-glucose, and collected in lysis buffer (0.025% SDS and 1% Triton X-100). The radioactivity was measured by liquid-scintillation counting.

### Macrophage Bacterial Killing Assay

The monolayer of macrophages (approximately 2 × 10^5^ cells) was infected with 2 × 10^6^ stationary phase of *S*. *aureus*. After 1 hour of infection, the extracellular bacteria were killed with gentomycin and lysostaphin, and removed. The macrophages were washed three times with PBS, then collected and lysed with 0.5% Triton X-100. The lysates were serially diluted with PBS for numeration of viable bacterial cells in macrophages.

### β-galactosidase Staining Assay

The Senescence β-galactosidase (β-gal) Staining Kit (Cell Signaling Technology) was used to detect the senescent cells. Briefly, macrophages were seeded in a six-well plate at the density of 1 × 10^6^ cells/plate. When cells confluence reached to around 75%, cells were washed once with PBS before incubated in fixative solution provided by Senescence β-gal Staining Kit for 15 min at room temperature. Cells were then rinsed twice with PBS and incubated with β-gal staining solution at 37 °C overnight. Next day, cells were observed under a microscope (100x magnification) for the development of blue color.

### Statistical Analysis

Results are expressed as means $\pm$ SEM. Statistical significance was evaluated using an unpaired, two-tailed Student’s t test was used. *P* < 0.05 was considered statistically significant.

## Electronic supplementary material

Supplementary Information
