# Supplementary material for: Down-regulation of guanylate binding protein 1 causes mitochondrial dysfunction and cellular senescence in macrophages
Source: Sci Rep. 2018 Jan 26;8:1679. doi: 10.1038/s41598-018-19828-7 (PMC5785964; doi:10.1038/s41598-018-19828-7)
Supplement: Supplementary file 1 — Supplementary Information [file 41598_2018_19828_MOESM1_ESM.pdf]

Down-regulation of guanylate binding protein 1 causes mitochondrial dysfunction and cellular senescence in macrophages

Xiaoxue Qiu[1], Hong Guo[1], Junshu Yang[2], Yinduo Ji[2], Chia-Shan Wu[3], and Xiaoli Chen[1*]

[1]Department of Food Science and Nutrition, and [2]Department of Veterinary Biomedical Sciences, College of Veterinary Medicine, University of Minnesota, Twin Cities, Minnesota, USA; [3]Department of Nutrition and Food Science, Texas A&M University, College Station, TX, USA

[*]**Corresponding author:** Dr. Xiaoli Chen, Department of Food Science and Nutrition, University of Minnesota-Twin Cities, St. Paul, MN 55108-1038, E-mail: xlchen@umn.edu

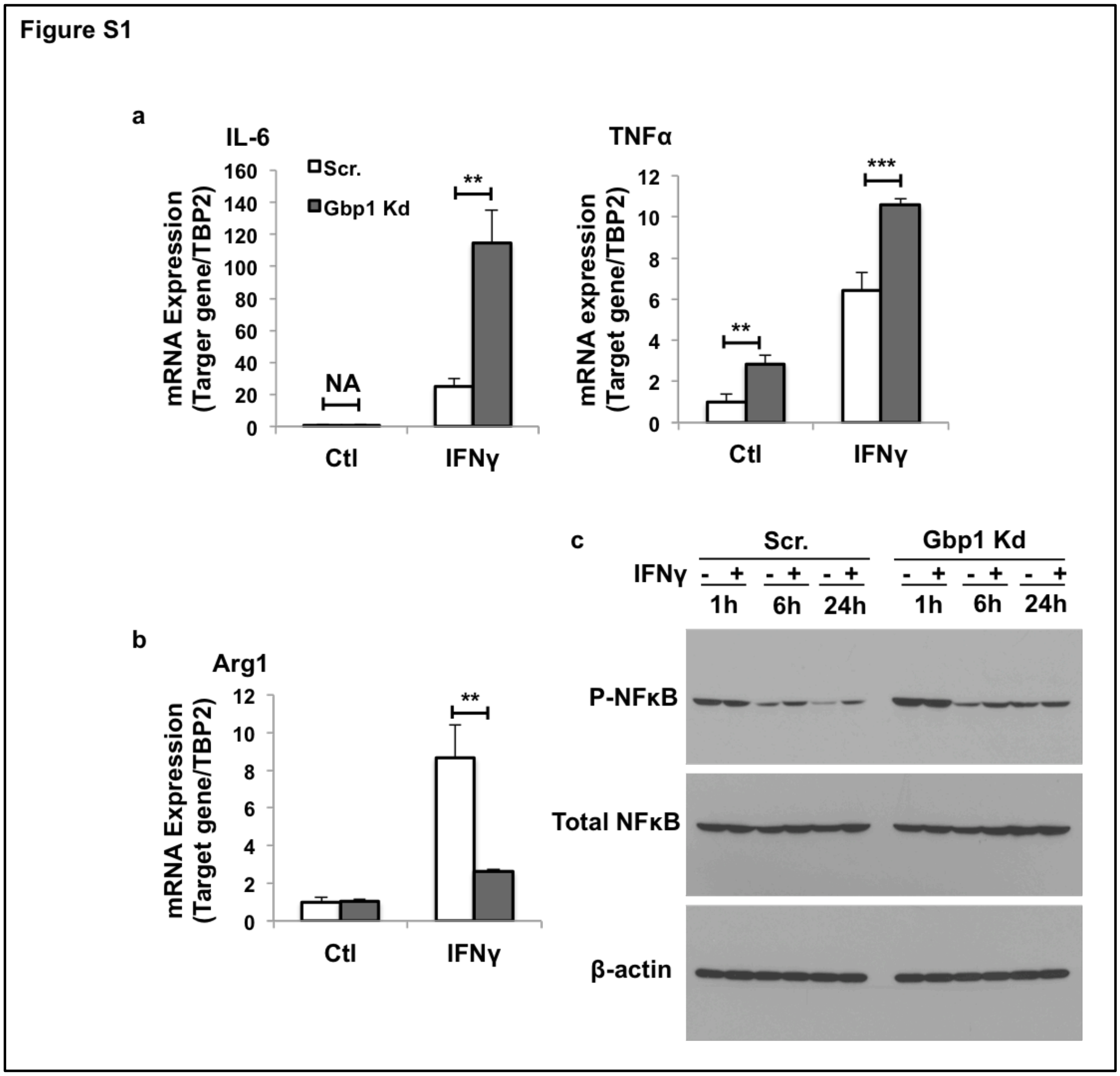

Figure S1. Effect of Gbp1 downregulation on M1 polarization of macrophages. (a-b) Scr and Gbp1 Kd macrophages were stimulated by IFNγ (10 ng/ml) for 24 hours. mRNA expression levels of pro-inflammatory (IL-6 and TNFα) and anti-inflammatory (Arg1) cytokines were determined by qPCR (n=3). Data are presented as mean ± SEM. ** $P < 0.01$, *** $P < 0.001$ versus Scr cells. (c) Macrophages were treated with IFNγ (10 ng/ml) for 1, 6 and 24 hours. NFκB Phosphorylated levels were detected by western blotting.

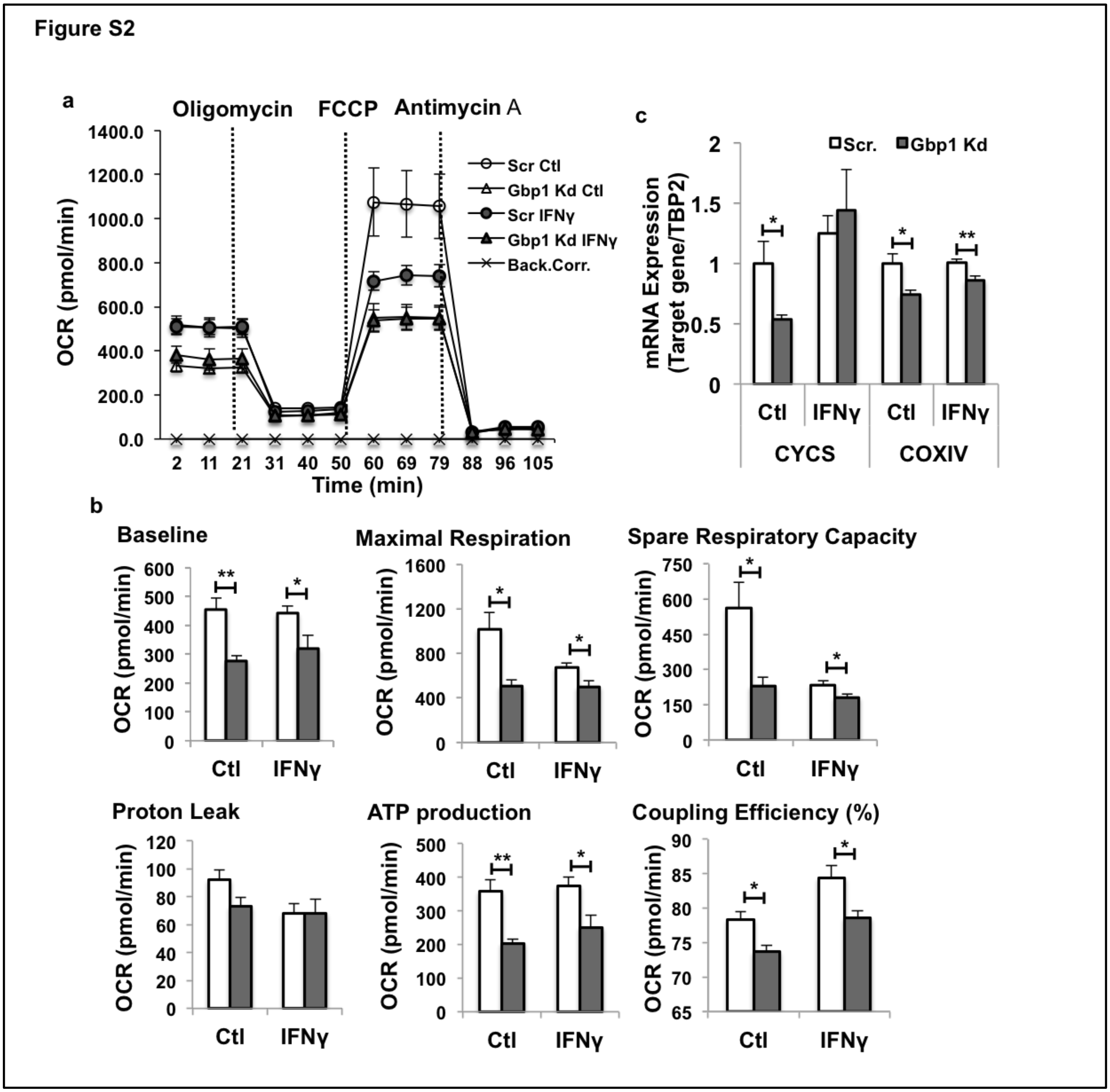

Figure S2. **Effect of Gbp1 downregulation on mitochondrial function in macrophages.** (a-b) OCR was determined in Scr and Gbp1 Kd macrophages treated with IFNγ (10 ng/ml) for 16 hours, n = 5. Back.Corr.: Background Correction. (c) mRNA expression levels of genes encoding electron transport chain components CYCS and COXIV were determined in Scr and Gbp1 Kd macrophages treated with 10 ng/ml IFNγ for 24 hours, n=3. Data are presented as mean ± SEM. * $P < 0.05$, ** $P < 0.01$ versus Scr cells.

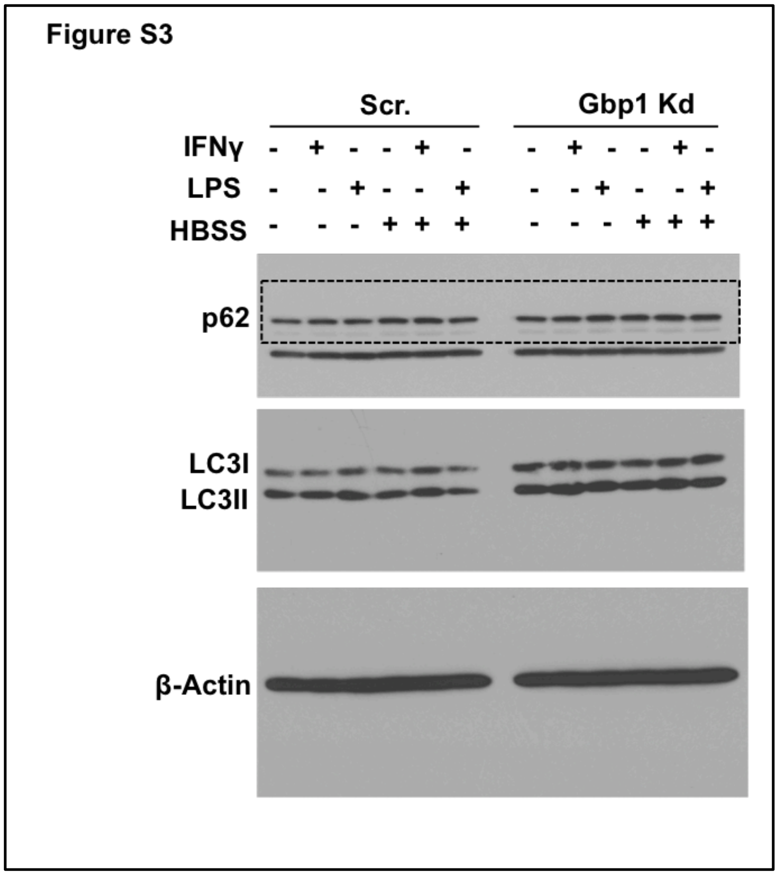

Figure S3. Autophagic flux in Gbp1 Kd macrophages. Macrophages were treated with LPS (1μg/ml) or IFNγ (10 ng/ml) for 15 min under fed or starvation conditions. Autophagic proteins p62 and LC3I/II were detected by western blotting.

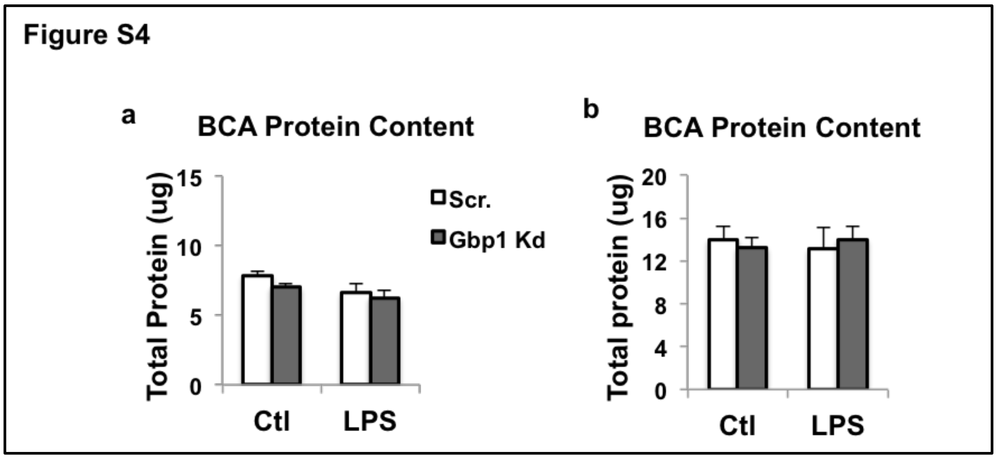

Figure S4. Total protein levels in Scr and Gbp1 Kd macrophages for cellular respiratory and glycolytic activity assay. (a-b) The BCA assay was performed to detect protein contents in Scr and Gbp1 Kd macrophages after cellular respiratory assay for OCR measurement (a) and glycolytic activity assay for ECAR measurement (b).

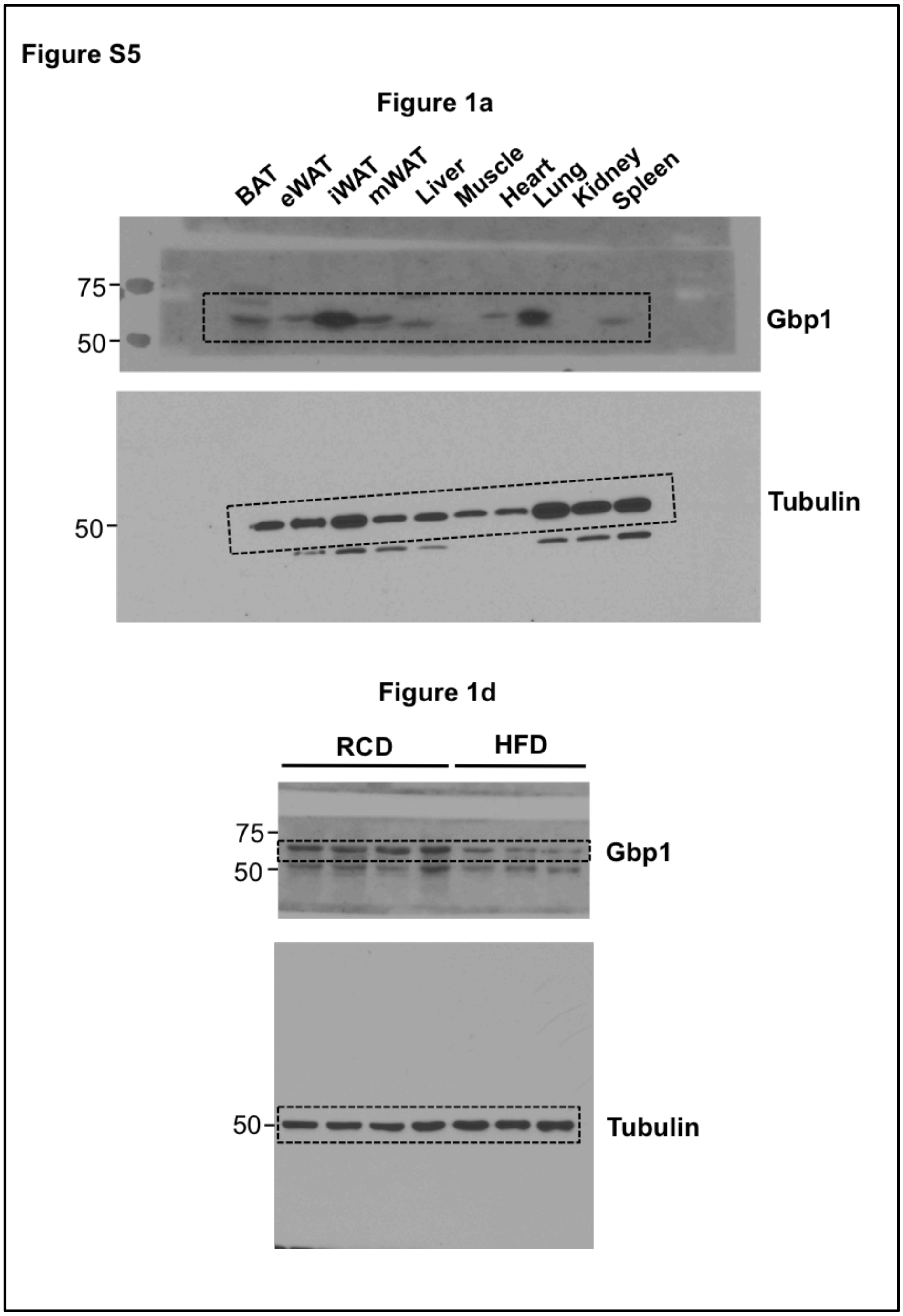

Figure S5. Unprocessed western blots for Fig. 1.

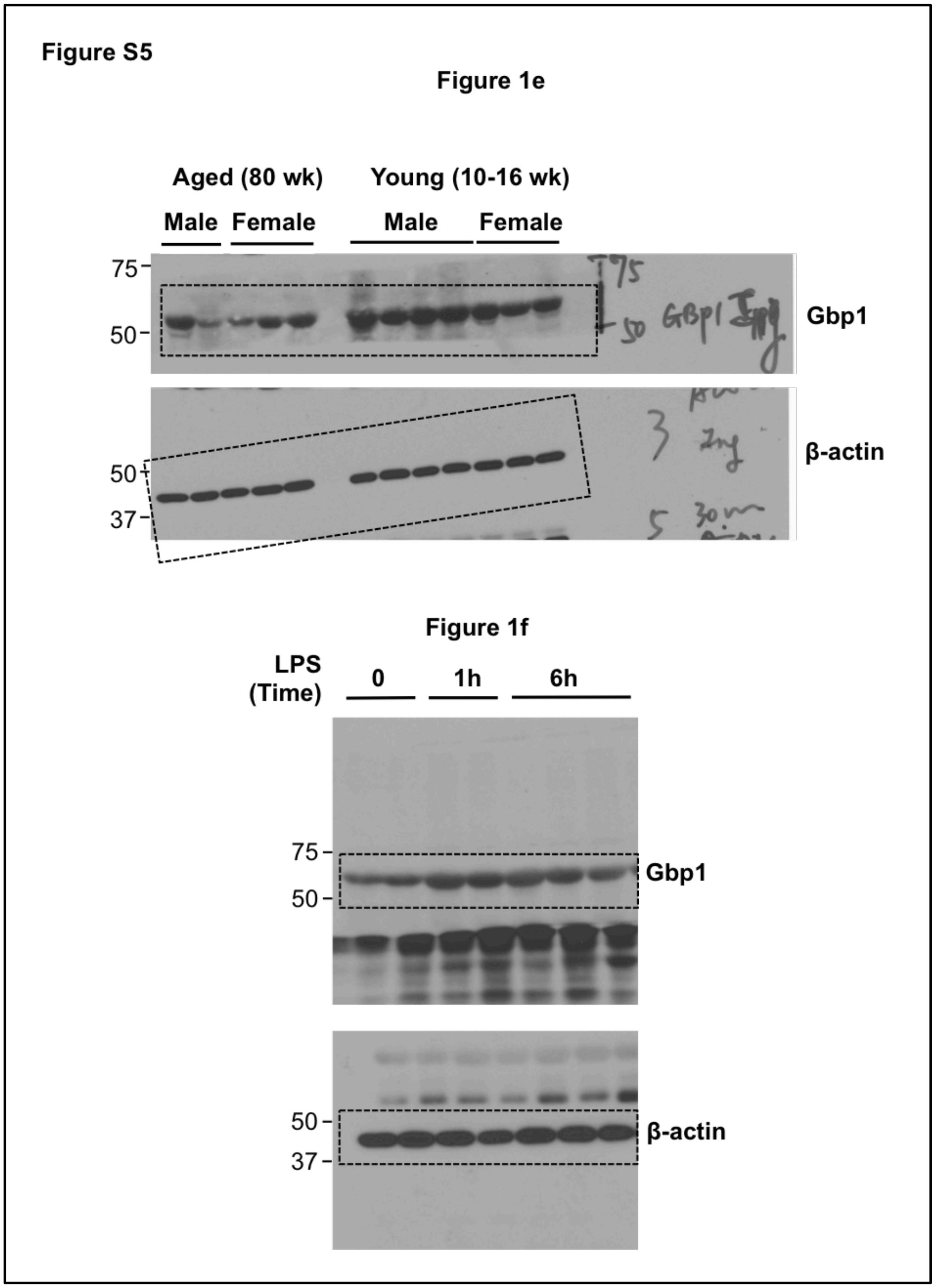

Figure S5. Unprocessed western blots for Fig. 1.

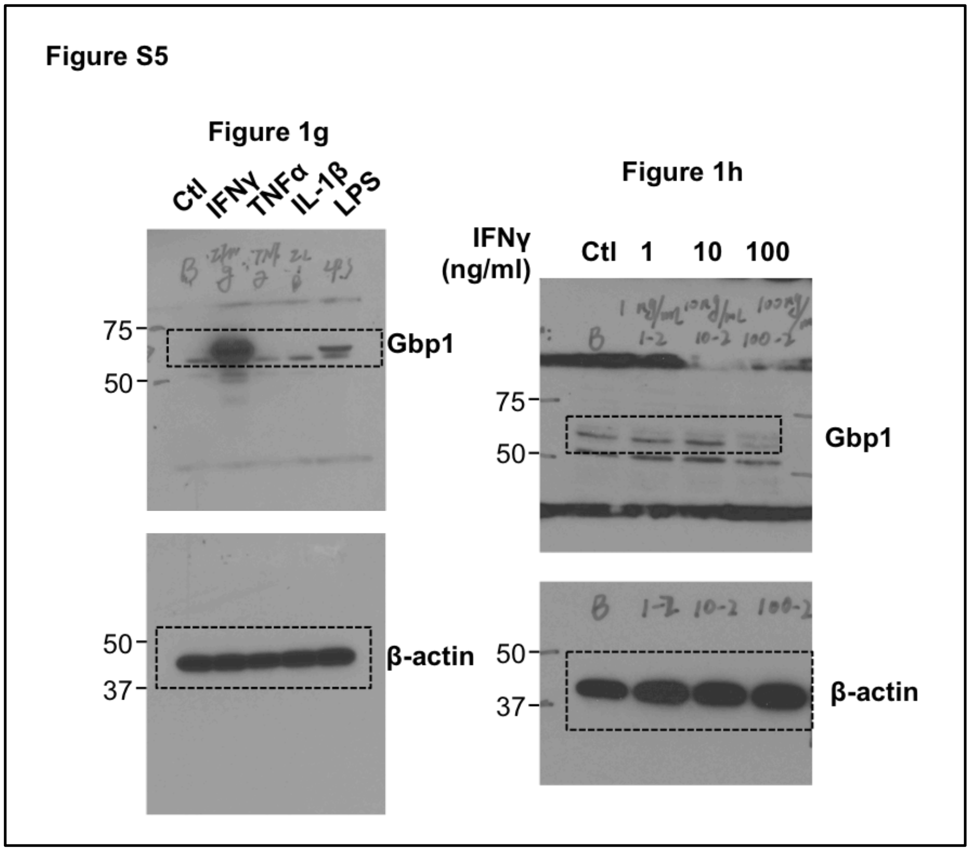

Figure S5. Unprocessed western blots for Fig. 1.

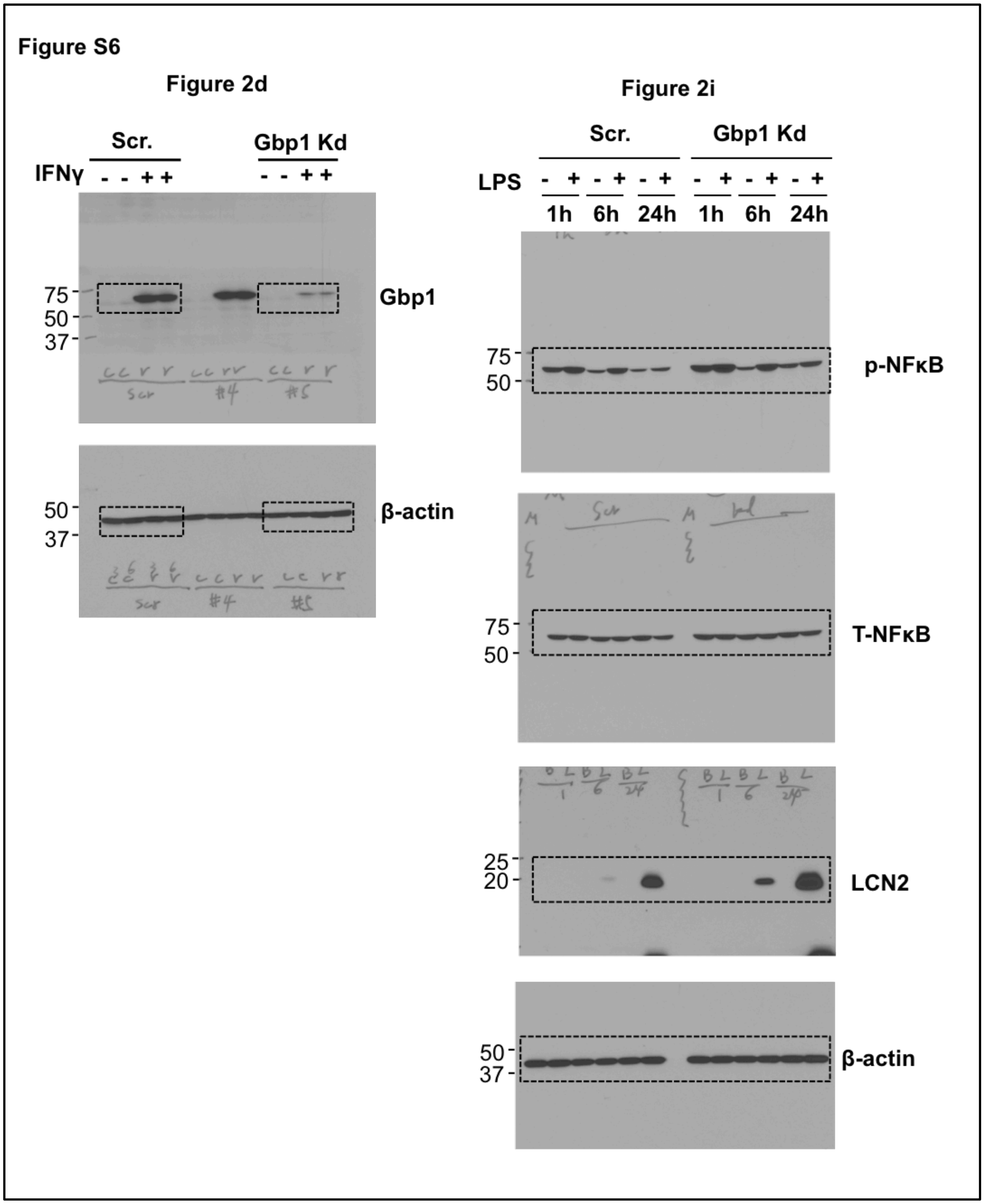

Figure S6. Unprocessed western blots for Fig. 2.

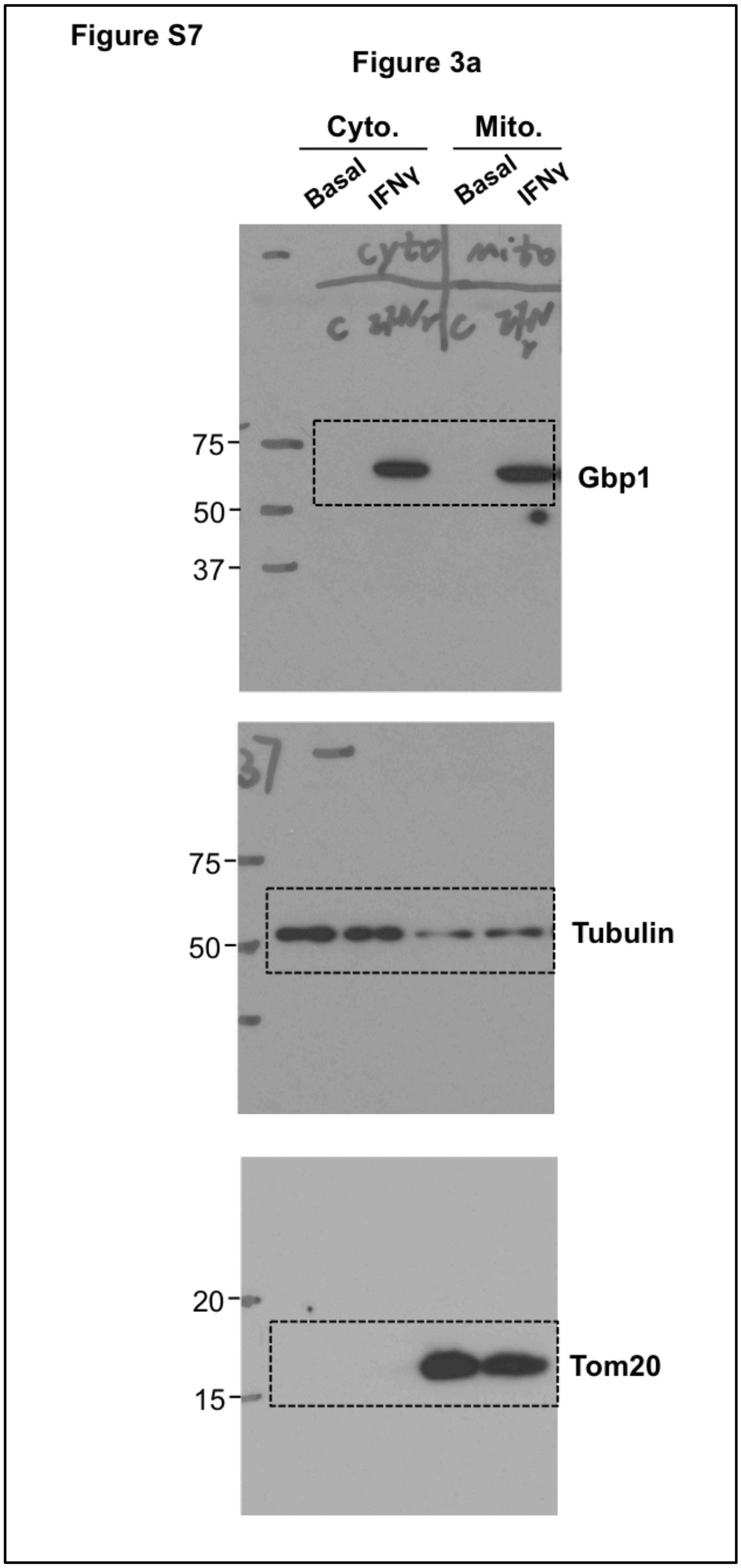

Figure S7. Unprocessed western blots for Fig. 3.

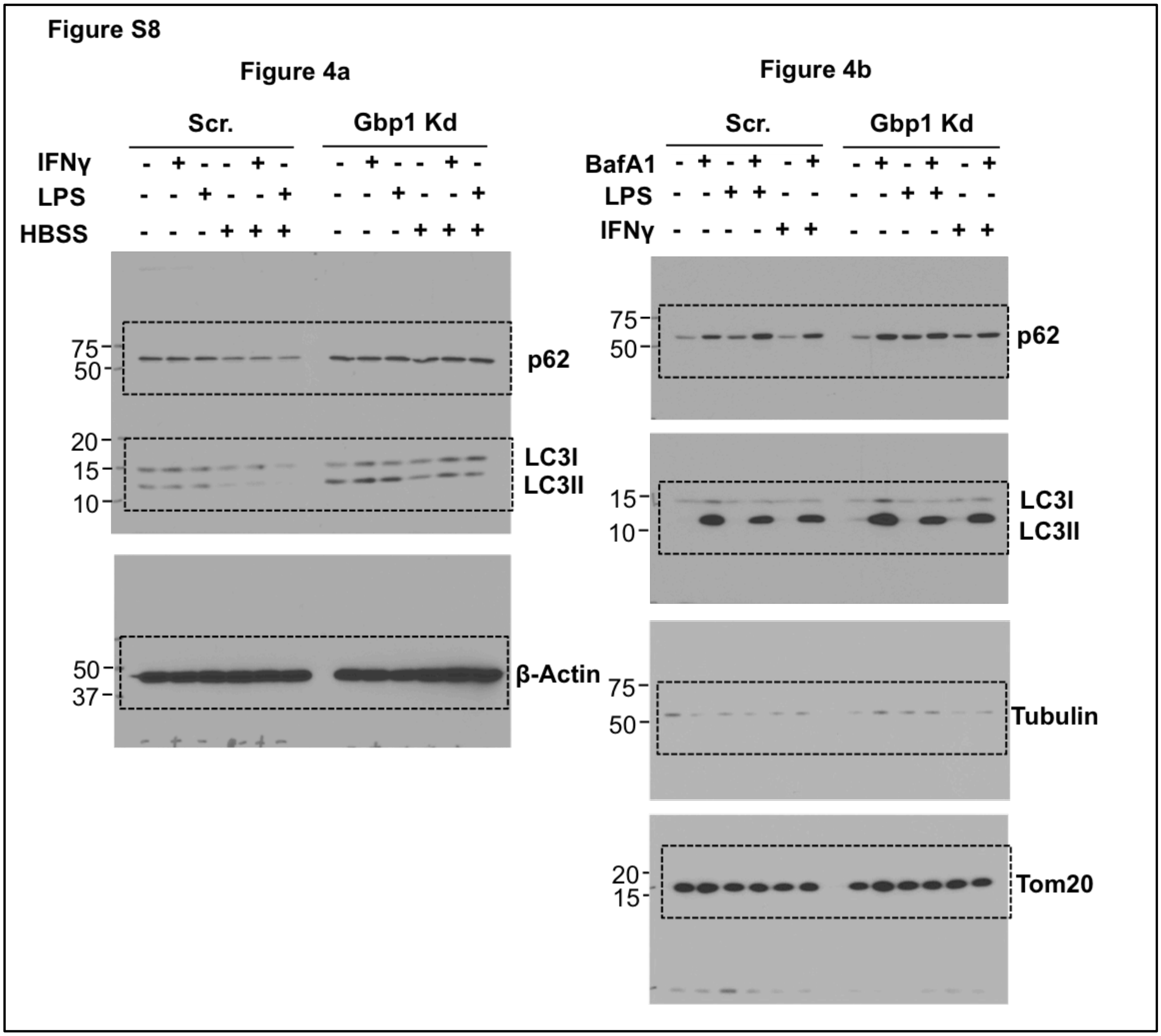

Figure S8. Unprocessed western blots for Fig. 4.

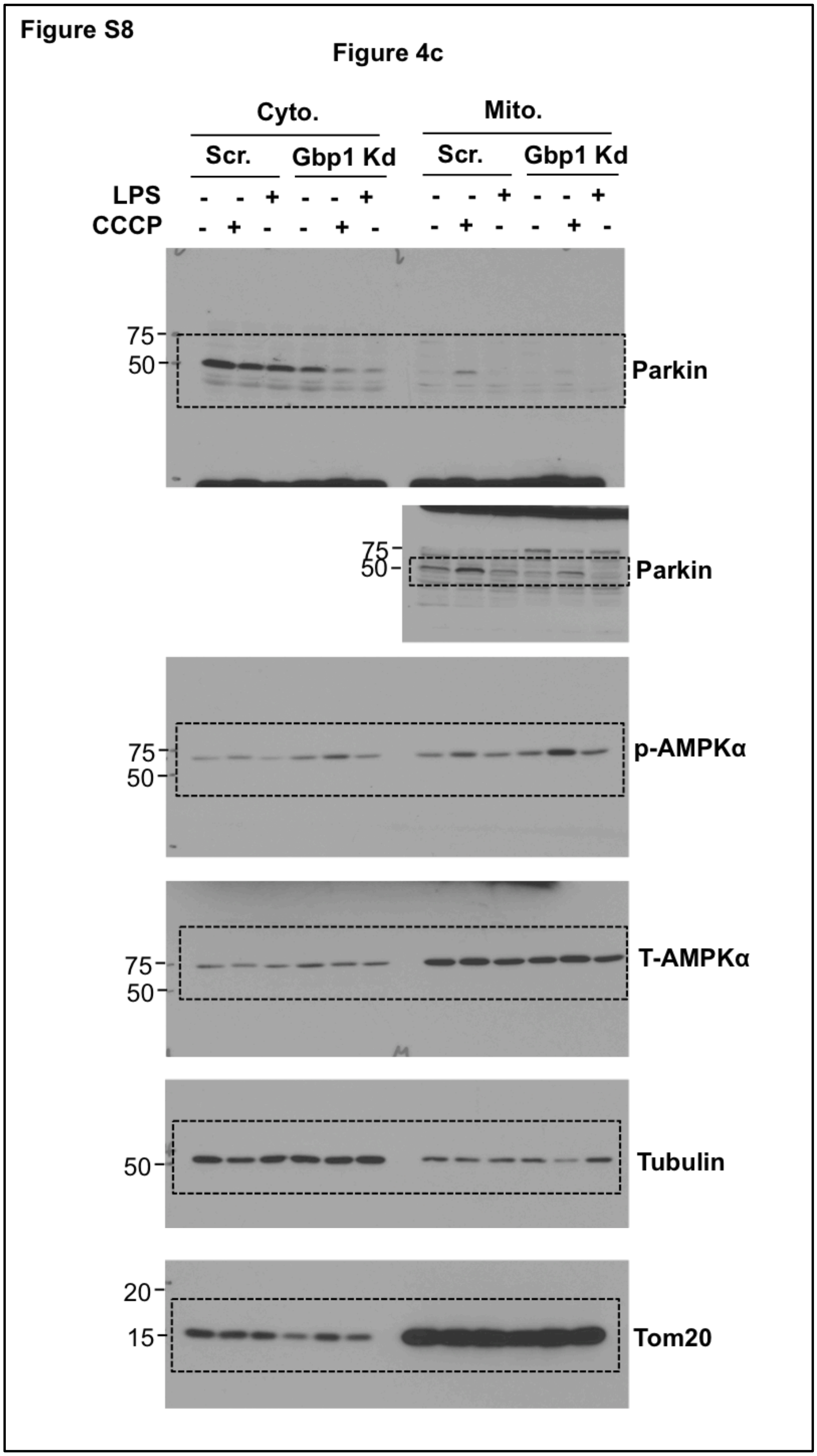

Figure S8. Unprocessed western blots for Fig. 4.

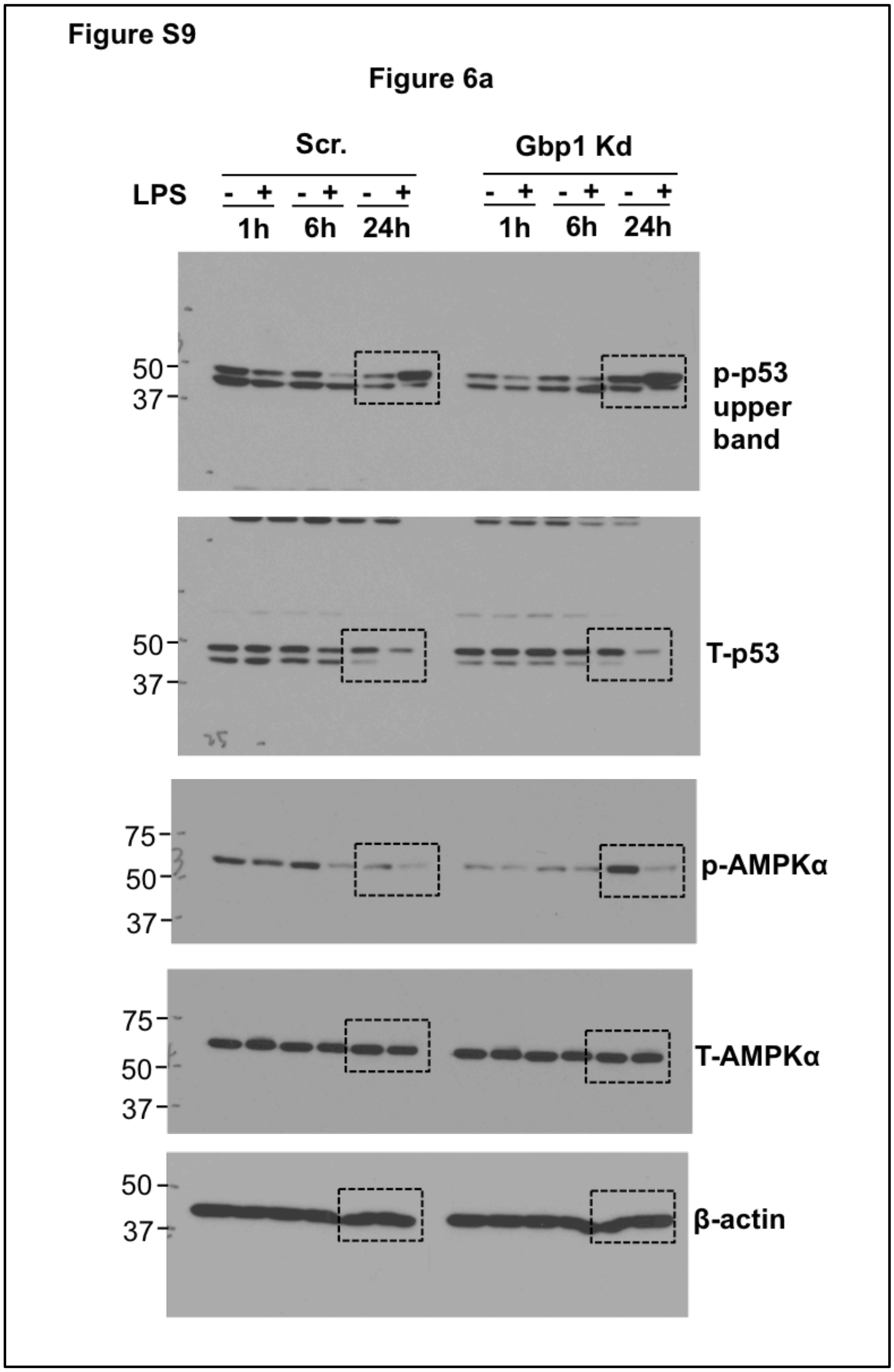

Figure S9. Unprocessed western blots for Fig. 6.

Table S1. Real-time qPCR primer sequences

Target	Forward primers (5′ to 3′)	Reverse primers (5′ to 3′)
Gbp1	AAGAACATGCCTCCACCTCG	ATCCAAAGCTGTCCCCGAAG
Gbp2	CCTGACCAGAGTGGGGTAGA	CAGTCGCGGCTCATTAAAGC
Gbp3	ACGGCAAGACCAAGACTCTG	GTCACTGCGTTCTCCAGACA
Gbp5	AGCCCAGGAAGAGGCTGATA	ATGGAGGGCTCAGGTTTTGG
Gbp7	AGTGGTGGTGGCCATTGTAG	ATCTCCTAAGCCCTCCGTGT
IL-6	TCTGCAAGAGACTTCCATCC	TTAGCCACTCCTTCTGTGAC
TNFα	CAGAAACACAAGATGCTGGG	CAAAAGAGGAGGCAACAAGG
COX2	CCTCCACTCATGAGCAGTCC	TCAGAGCATTGGCCATAGAA
Arg1	AACACGGCAGTGGCTTTAACC	GGTTTTCATCTGGCGCATTC
CYCS	CACGGCTCTCCCTTTCTCAAG	ACAGTTGCCTCCTGGTGGTTA
COXIV	ATGTCACGATGCTGTCTGCC	GTGCCCCTGTTCATCTCGGC
CPT1b	CCTCCCTGGGCATGATTG	ACGCCACTCACGATGTTCTTC
Acox1	GTGCAGCTCAGAGTCTGTCCAA	TACTGCTGCGTCTGAAAATCCA
CS	GTTGGCAAAGACGTGTCAGAT	TCAGAGCAAACTCTCGCTGAC
Gpx1	CCGCTTTCGTACCATCGACAT	CCAGTAATCACCAAGCCAATGC
GSTA4	CTGTACTGTCCGACTTCCCTC	CTCTGACTTCCGGGTTGCAG
SOD1	AAGCATGGCGATGAAAGCG	ACAACACAACTGGTTCACCGC
Prdx1	AGTCCAGGCCTTCCAGTTCACT	GGCTTGATGGTATCACTGCCAG
UCP2	TGACAAACAGGTCAAGAGAGGGCA	TCAGGCCAACTGACAGCATTCCTA
Glut1	TCAACGAGCATCTTCGAGAAGGCA	TCGTCCAGCTCGCTCTACAACAA
GYS1	TTCTACAACAACCTGGAG	CTGAGCAGATAGTTGAGC
Tbp2	GAAGAACAATCCAGACTAGCAGCA	CCTTATAGGGAACTTCACATCACAG
